# Glycogen Synthase Kinase 3 Regulates the Genesis of Displaced Retinal Ganglion Cells3

**DOI:** 10.1523/ENEURO.0171-21.2021

**Published:** 2021-10-05

**Authors:** Elena Kisseleff, Robin J. Vigouroux, Catherine Hottin, Sophie Lourdel, Leah Thomas, Parth Shah, Alain Chédotal, Muriel Perron, Anand Swaroop, Jerome E. Roger

**Affiliations:** 1 Paris-Saclay Institute of Neuroscience, CERTO-Retina France, CNRS, Université Paris-Saclay, 91400, Saclay, France; 2 Neurobiology-Neurodegeneration and Repair Laboratory, National Eye Institute, National Institutes of Health, Bethesda, Maryland 20892; 3 UPMC Université Paris 06, INSERM, CNRS, Institut de la Vision, Sorbonne Universités, 75012 Paris, France

**Keywords:** cell death, displaced ganglion cells, glycogen synthase kinase 3, post-translational modifications, medial terminal nucleus, retinal development

## Abstract

Glycogen synthase kinase 3 (GSK3) proteins (GSK3α and GSK3β) are key mediators of signaling pathways, with crucial roles in coordinating fundamental biological processes during neural development. Here we show that the complete loss of GSK3 signaling in mouse retinal progenitors leads to microphthalmia with broad morphologic defects. A single wild-type allele of either *Gsk3α* or *Gsk3β* is able to rescue this phenotype. In this genetic context, all cell types are present in a functional retina. However, we unexpectedly detected a large number of cells in the inner nuclear layer expressing retinal ganglion cell (RGC)-specific markers (called displaced RGCs, dRGCs) when at least one allele of *Gsk3α* is expressed. The excess of dRGCs leads to an increased number of axons projecting into the ipsilateral medial terminal nucleus, an area of the brain belonging to the non-image-forming visual circuit and poorly targeted by RGCs in wild-type retina. Transcriptome analysis and optomotor response assay suggest that at least a subset of dRGCs in *Gsk3* mutant mice are direction-selective RGCs. Our study thus uncovers a unique role of GSK3 in controlling the production of ganglion cells in the inner nuclear layer, which correspond to dRGCs, a rare and poorly characterized retinal cell type.

## Significance Statement

Glycogen synthase kinase 3 (GSK3) proteins (GSK3α or GSK3β) are key mediators of signaling pathways, especially in the CNS but poorly described in the retina. Here we show that the complete loss of GSK3 in mouse retinal progenitors leads to microphthalmia. However, when only one allele of *Gsk3α* or *Gsk3β* is present, all cell types are present with a functional retina. Interrestingly, we unexpectedly uncovered a unique role of GSK3s in controlling the genesis of retinal ganglion cells in the inner nuclear layer, which could correspond to a rare and poorly characterized retinal cell type. Therefore, our mouse models potentially offer a unique and powerful model system to study the visual function of displaced retinal ganglion cells in mammals.

## Introduction

Glycogen synthase kinase 3α (GSK3α) and β (GSK3β) are functionally redundant serine/threonine kinases encoded by two different genes, sharing 95% identity in their kinase domain (Doble et al., 2007). GSK3s exist at the crossroads of multiple signaling pathways and act as a key molecular switch to mediate their output and guide distinct cellular processes (Doble and Woodgett, 2003; Espinosa et al., 2003; Wang and Li, 2006; Shimizu et al., 2008; Jin et al., 2009; Cole, 2012). Among the signaling pathways regulated by GSK3s, the Wnt canonical pathway is the most well described, with GSK3β inhibition triggering an increase in *β*-catenin protein levels and its nuclear translocation to activate target gene expression (Doble and Woodgett, 2003).

GSK3 is a key regulator of neural stem/precursor cell proliferation in developing as well as adult brain (Eom and Jope, 2009; Kim et al., 2009; Hur and Zhou, 2010; Pachenari et al., 2017). Conditional deletion and gain-of-function experiments indicate that GSK3 promotes neuronal differentiation (Kim et al., 2009; Hur and Zhou, 2010). GSK3 exerts its effects through the phosphorylation of key proteins involved in neural development, including proneural factors such as Neurogenin 2 and NeuroD (Moore et al., 2002; Li et al., 2012). In addition, GSK3 fine-tunes the balance between cell death and survival, and its altered function is associated with neurodegenerative pathologies including Alzheimer’s disease, bipolar disorders, and Parkinson’s disease (Kremer, 2011; Medina et al., 2011; Jacobs et al., 2012; Li et al., 2014; Maurer et al., 2014; Golpich et al., 2015).

GSK3 proteins are widely expressed in the developing retina (Pérezleón et al., 2013). GSK3-dependent phosphorylation was shown to control the timing of proneural factor activity and thereby regulate retinal cell fate determination. For instance, the inhibition of GSK3 signaling in the developing *Xenopus* retina leads to increase in early-born cell types at the expense of late-born cells (Marcus et al., 1998; Moore et al., 2002).

To elucidate GSK3 function in mammalian retina development, we generated conditional loss-of-function alleles of *Gsk3α* and *Gsk3β* in retinal progenitor cells (RPCs). We showed that complete loss of both GSK3s severely impacts retinal morphology with microphthalmia phenotype, which could be completely rescued with the expression of just one *Gsk3α* or *Gsk3β* wild-type (WT) allele. We also noted the presence of a large number of displaced retinal ganglion cells (dRGCs) in the inner nuclear layer (INL) in the absence of either *Gsk3α* or *Gsk3β.* In normal conditions, this is a rare retinal cell subtype, poorly characterized so far. Anterograde labeling of the axonal ganglion cell projections into the brain of *Gsk3* mutant mice, allowed us to further support their dRGCs identity. Our study thus identifies GSK3 as a possible determinant of dRGC genesis. We also provide transcriptomic data and visual tests, suggesting that at least a subset of these supernumerary dRGCs in *Gsk3* mutant retinas are direction-selective RGCs.

## Materials and Methods

### Animals and tissue collection

All animal experiments have been conducted in accordance with the European Communities Council Directive of 22 September 2010 (2010/63/EEC), the effective European Union guidelines, and the Association for Research in Vision and Ophthalmology statement for the use of animals in ophthalmic and visual research. All animal care and experimentation were also conducted in accordance with guidelines, under license APAFIS#1018–2016072611404304 granted by Institutional Animal Care Committee 059 in France and by the Animal Care and Use Committee at the National Institutes of Health (license ASP#650). *Gsk3α* and *Gsk3β* floxed mice were generously provided by Jim Woodgett (University of Toronto, Toronoto, ON, Canada). Floxed *Gsk3* mice were mated with those carrying the retina-specific regulatory element of murine *Pax6* driving the expression of the Cre recombinase (*α-Cre*) in retinal progenitors as early as embryonic day 10.5 (E10.5) generously provided by Peter Gruss, Max-Planck-Institute of Biophysical Chemistry, Göttingen, Germany (Marquardt et al., 2001). Mice are on a mixed background C57BL/6J and 129/SvJ. Animals from either sex were used for experimental procedures. All mouse genotyping was performed as described previously (Hamon et al., 2019).

### Hematoxylin and eosin staining and immunostaining

Methacrylate sections were used for hematoxylin and eosin (H&E) staining as previously described (Hamon et al., 2019). For immunohistochemistry (IHC) on frozen sections, enucleated eyeballs were fixed at the required stage in 4% PFA for 60 min on ice and incubated in increasing concentrations of sucrose (10%, 20%, and 30%), then embedded in Optimal Cutting Temperature compound. Embedded eyeballs were serially cut into 12 μm sections using a cryostat. For embryonic stages, pregnant females were sacrificed and whole heads of pups were harvested in paraffin. IHC was performed as described previously (Roger et al., 2012). Primary and secondary antibodies are listed in Table 1. Sections were counterstained with 1:1000 DAPI (4′,6′-diamidino-2-phenylindole dihydrochloride; 1 mg/ml; Thermo Fisher Scientific).

**Table 1:** List of primary and secondary antibodies used for immunohistochemistry and western blot

Antigen	Host	Supplier	Catalog no.	Dilution	
				IHC	WB
Primary antibodies					
α-Tubulin	Mouse	Sigma-Aldrich	T5168		1:200.000
GSK3α/β	Mouse	Thermo Fisher Scientific	44–610	1:250	1:1000
GSK3β	Mouse	BD	610201	1:250	
Brn3a	Mouse	Santa Cruz Biotechnology	sc-8429	1:200	
Calbindin D-28k	Rabbit	Swant	300	1:100	
Calretinin	Mouse	EMD Millipore	MAB1568	1:1000	
Cone arrestin	Rabbit	EMD Millipore	AB15282	1:1000	
Rhodopsin	Mouse	Abcam	MAB5316	1:2000	
Tbr2	Rat	Thermo Fisher Scientific	14–4876	1 :300	
Foxp2	Goat	Santa Cruz Biotechnology	sc-21069	1 :1000	
Rbpms	Rabbit	PhosphoSolutions	1830-RBPMS	1 :400	
CHAT	Goat	Millipore	AB144P	1 :100	
Secondary antibodies					
Alexa Fluor-555 anti-mouse IgG2A	Goat	Thermo Fisher Scientific	A21127	1:1000	
Alexa Fluor-555 anti-mouse IgG2B	Goat	Thermo Fisher Scientific	A21147	1:1000	
Alexa Fluor-488 anti-rabbit	Donkey	Thermo Fisher Scientific	A21206	1:1000	
Alexa Fluor-488 anti-mouse IgG1	Goat	Thermo Fisher Scientific	A21240	1:1000	
Alexa Fluor-488 anti-rabbit	Goat	Thermo Fisher Scientific	A21244	1:1000	
HRP anti-mouse IgG	Goat	Sigma-Aldrich	A4416	1:5000	
Alexa Fluor-488 anti-goat	Donkey	Thermo Fisher Scientific	A11055	1:1000	

WB, Western blot; IHC, Immunohistochenistry.

### 5-Ethynyl-2′-deoxyuridine labeling and terminal deoxynucleotidyl transferase-mediated biotinylated UTP nick end labeling assay

For 5-ethynyl-2′-deoxyuridine (EdU) labeling, females were injected intraperitoneally with 10 mm of EdU (Thermo Fisher Scientific). EdU incorporation was detected on paraffin sections or frozen sections using the Click-iT EdU Imaging Kit following manufacturer recommendations (Thermo Fisher Scientific). Apoptosis was detected by TUNEL (terminal deoxynucleotidyl transferase-mediated biotinylated UTP nick end labeling) assays using *in situ* cell death detection kit (Promega). All images were acquired using a confocal microscope (model LSM710, Zeiss) and Zen software (Zeiss).

### Immunoblotting

Frozen retinas were lysed by sonication in lysis buffer (20 mm Na_2_HPO_4_, 250 mm NaCl, 30 mm NaPPi, 0.1% NP-40, 5 mm EDTA, 5 mm DTT) supplemented with protease inhibitor cocktail (Sigma-Aldrich). Lysates concentration was determined using a Lowry protein assay kit (BIO-RAD) following sonication and centrifugation. Protein supernatants were separated under denaturing conditions by SDS-PAGE, transferred onto nitrocellulose membrane, and probed with antibodies (Table 1), as described (Roger et al., 2010). Proteins were visualized using enhanced chemiluminescence kit (BIO-RAD). α-Tubulin was used for normalization. Quantification was performed using ImageJ software (http://imagej.nih.gov/ij/; provided in the public domain by NIH).

### Retinal flat mount

Fixed retinas were permeabilized and blocked in a solution containing 0.5% Triton X-100, 5% normal donkey serum, 1× PBS, 0.1 g/L thimerosal for 1 d at room temperature (RT) under agitation. Primary antibodies were diluted in a solution containing 0.5% Triton X-100, 5% normal donkey serum, 10% dimethylsulfoxide, 1× PBS, and 0.1 g/L thimerosal for 3 days at RT under agitation. The retinas were then washed for 1 d in PBST (1× PBS, 0.5% Triton X-100). Secondary antibodies were diluted in the same solution as primary antibodies and left for 2 d. After retinas were washed for 1 d, they were flat-mounted on slides and imaged using a scanning confocal microscope (model FV1000, Olympus). Primary and secondary antibodies are listed in Table 1.

### Electroretinography

Electroretinogram (ERG) recordings were performed using a focal ERG module attached to a retinal imaging microscope (model Micron IV, Phoenix Technology Group). Briefly, mice were dark adapted overnight and prepared for the experiment under dim red light. The mice were anesthetized with ketamine (100 mg/kg) and xylazine (10 mg/kg) and received topical proparacaine hydrochloride (0.5%; Alcon) via eye drops. Pupils were dilated with tropicamide (1%; Alcon) and phenylephrine (2.5%; Alcon) and were lightly coated with GONAK hypromellose ophthalmic demulcent solution (2.5%; Akorn). The lens of the Micron IV was placed directly on the cornea, and a reference electrode was placed on the mouse head. Scotopic responses were elicited with a series of flashes of increasing light intensities from −1.7 to 2.2 cd/s/m^2^. Photopic responses were elicited under rod-desensitizing background light with a series of flashes of increasing light intensities from −0.5 to 2.8 cd/s/m^2^. Values of a- and b-wave were extracted and plotted for comparisons between groups of interest.

### Optomotor response

Real-time video tracking and automated measurements of compensatory head movements in freely moving mice were performed using an optomotor response (OMR) recording setup (PhenoSys; Kretschmer et al., 2013, 2015). Each mouse was placed on a platform in the center of four computer-controlled LCD monitors. Visual stimuli were sinusoidally modulated luminance gratings generated by four LCD screens (60 Hz refresh rate; OkrArena, PhenoSys), presented with a constant rotation. Video tracking considered the distance of the animal from the monitors, thereby keeping the spatial frequency of the retinal image constant and providing data for automated OMR quantifications.

OMRs were recorded using two different Michelson contrasts and different spatial frequencies (presented in random order) in the following two mouse groups: 100% contrast or 50% contrast (*n* = 13 for *Gsk3α^f/+^β^f/f^* and 18 for *Gsk3α^f/+^β^f/f^*;*α-Cre* genotype). All stimuli were presented for 60 s randomly in either clockwise or counterclockwise direction. The measurements were completed in three trials for each animal. At 100% and 50% contrast, OMRs were recorded in response to sinusoidal gratings at 12 spatial frequencies between 0.0125 and 0.5 cycles per degree (cpd). The number of head movements recorded at a speed range from 2 to 14°/s in the same direction as the stimulus (*T*_Correct) and in the opposite direction (*T*_Incorrect) were used to calculate the OMR indices (*T*_Correct/*T*_Incorrect) at each spatial frequency.

### Retrograde labeling of retinal ganglion cells

For retrograde labeling, eyes were enucleated with a piece of the optic nerve and fixed in 4% PFA for 30 min. Rhodamine B isothiocyanate–dextran (Sigma-Aldrich) was applied on the top of the optic nerve and incubated for 60 min. Eyes were flat-mounted after the remaining dye was washed out for 48 h in PBS at 4°C. The *z* series images were acquired using a confocal microscope (model SP5, Leica Biosystems), and 3D reconstruction was performed using Volocity (PerkinElmer).

### Anterograde labeling of retinal ganglion cell projections

#### Anterograde labeling

For anterograde tracing of retinal projections, a cholera toxin β subunit (CTB) was used. Animals were anesthetized using a cocktail of ketamine (60 mg/kg) and xylazine (10 mg/kg), and a subsequent bilateral injection of 1.2 μl of CTB at 1 mg/ml coupled with either Alexa Fluor-555 or Alexa Fluor-647 (Beckman Coulter Life Sciences) was performed intravitreally. Three days following the injection, mice were perfused with 4% PFA.

#### Tissue clearing and 3D imaging

For 3D imaging of CTB-labeled brains, a methanol clearing protocol was conducted using modification from the iDISCO+ protocol (Belle et al., 2014, 2017). Briefly, brains were dehydrated by immersion in progressive baths of methanol/1× PBS (20%, 40%, 60%, 80%, 100%, 100%) for 2 h each at RT on a tube rotator (model SB3, Stuart) at 14 rpm, using a 15 ml centrifuge tube (model TPP, Dutcher) protected from light. Following these baths, samples were immersed overnight in two-thirds cubic meter dichloromethane (DCM; Sigma-Aldrich) and then were given a 30 min bath in 100% DCM before being transferred in dibenzyl ether (Sigma-Aldrich) overnight prior imaging.

3D imaging/image acquisition for all samples was performed as described previously (Belle et al., 2014, 2017). Acquisitions were performed using a microscope (UltraMicroscope I, LaVision BioTec) with ImspectorPro software (LaVision Biotec). The step size between each image was fixed at 2 μm with a numerical aperture of 0.120 and 150 ms acquisition using a PCO Edge SCMOS CCD camera (2560 × 2160 pixel size; LaVision BioTec).

#### Image analysis

Imaris x64 software (version 9.1.2; Bitplane) was used for all image analysis. Stack images were first converted from .tiff to .ims files using the Imaris file converter version 9.1.2. 3D reconstruction was visualized with the “volume rendering” function. To isolate ipsilateral and contralateral medial terminal nucleus (MTN) volumes, manual segmentation was conducted using the “surface” tool and the isoline selection (density, 10%). Each ipsilateral and contralateral projection of the MTN was segmented to generate a volume (cubic micrometer). Movie reconstruction with .tiff series was performed with ImageJ (1.50e, Java 1.8.0_60, 64-bit) and iMovie (version 10.1.1).

### Whole-transcriptome sequencing and analysis

Whole-transcriptome sequencing was performed on three independent biological replicates from *Gsk3α^f/+^β^f/f^;α-Cre* and *Gsk3α^f/+^β^f/f^* retinas at postnatal day 60 (P60). After harvesting, both retinas for each animal were immediately frozen. RNA was extracted using a Nucleospin RNA Plus Kit (Macherey-Nagel). RNA quality and quantity were evaluated using a BioAnalyzer 2100 with RNA 6000 Nano Kit (Agilent Technologies). Stranded RNA sequencing (RNA-Seq) libraries were constructed from 100 ng high-quality total RNA (RNA integrity number, >8) using the TruSeq Stranded mRNA Library Preparation Kit (Illumina). Paired-end sequencing of 40 base lengths was performed on a NextSeq 500 system (Illumina). Pass-filtered reads were mapped using STAR and aligned to mouse reference genome GRCm38.94 (Dobin et al., 2013). A count table of the gene features was obtained using FeatureCounts (Liao et al., 2014). Normalization, differential expression analysis and FPKM (fragments per kilobase of exon per million fragments mapped) values were computed using EdgeR (Chen et al., 2014). An FPKM filtering cutoff of 1 in at least one of the six samples was applied. A false discovery rate (FDR) of ≤0.05 was considered significant and a fold change (FC) cutoff of 1.5 was applied to identify differentially expressed genes (DEGs). Comprehensive gene list analysis, enriched biological pathways, and gene annotation were based on the Gene Ontology classification system using Metascape (Zhou et al., 2019). Data visualization was done using the GOplot R package (Walter et al., 2015). To evaluate the expression of the DEGs in RGCs, we used published whole-transcriptome analysis from purified RGCs that are available on the Gene Expression Omnibus database (GSE87647; Sajgo et al., 2017).

### Gene expression analysis by real-time PCR

After RNA extraction using the Nucleospin RNA Plus Kit (Macherey-Nagel), 500 ng of total RNA was reverse transcribed using the iScript cDNA Synthesis Kit according to manufacturer instructions (BIO-RAD). Primers used for quantitative real-time PCR (qRT-PCR) are shown in Table 2. For each qRT-PCR, 2 μl of a 10-fold dilution of synthetized cDNA was used, and the reactions were performed in technical triplicates on a C1000 thermal cycler (model CFX96 Real-Time System, BIO-RAD) using SsoFast EvaGreen Supermix (BIO-RAD) as previously described (Hamon et al., 2019). qRT-PCR experiments were performed on three independent biological replicates. Differential expression was determined using the ΔΔCt method with the geometric average of *Rps26* and *Srp72* as an endogenous control (Livak and Schmittgen, 2001).

**Table 2:** List of primers used for qRT-PCR

Gene name	Primer forward	Primer reverse
*Cartpt*	5′-TAAAGTTTGCGTTCCCCTCAG-3′	5′-CAACACCATTCGAGGCATTCT-3′
*Th*	5′-ACTATGCCTCTCGTATCCAGC-3′	5′-CGGATGGTGTGAGGACTGTC-3′
*Epha2*	5′-GACCTCCCCATCTTCATTTGG-3′	5′-GCGTACAGTGCCCTAGTCATA-3′
*Cplx1*	5′-GGTGATGAGGAAAAGGACCCC-3′	5′-TCTTGGCGTACTTTGCTTTGC-3′
*Chrna5*	5′-CTTGAGTACCAACACTGTCCG-3′	5′-CCAGTACTCCAAAGATGCCCT-3′
*Chrna2*	5′-CATTATCGTCTGCTTCCTGGG-3′	5′-CTTGGAGCCAACATGAGGGA-3′
*Chrna7*	5′-CTGTAGCTGTCGGTCTTGAGA-3′	5′-CAATGATATGCCGGTGATGGG-3′
*Chrnb4*	5′-AAACTGATCTGGCTACCTCCC-3′	5′-GTAGAGAGTCCAGGAGATGCC-3′

### Statistical analysis

Statistical analyses were performed with GraphPad Prism version 8.3.0 (GraphPad Software). Results are reported as the mean ± SEM. A nonparametric Mann–Whitney *U* test was used to analyze cell counting and qPCR data. A *p* value of ≤0.05 was considered significant. For OMR assay statistical analysis, a Grubbs’ test was performed at 5% to remove outliers followed by two-way ANOVA (genotype and spatial frequency) with Bonferroni *post hoc* test. A *p* value ≤0.05 was considered significant.

## Results

### Retinal progenitor-specific deletion of both *Gsk3α* and *Gsk3β* results in microphthalmia

**Figure 1.**
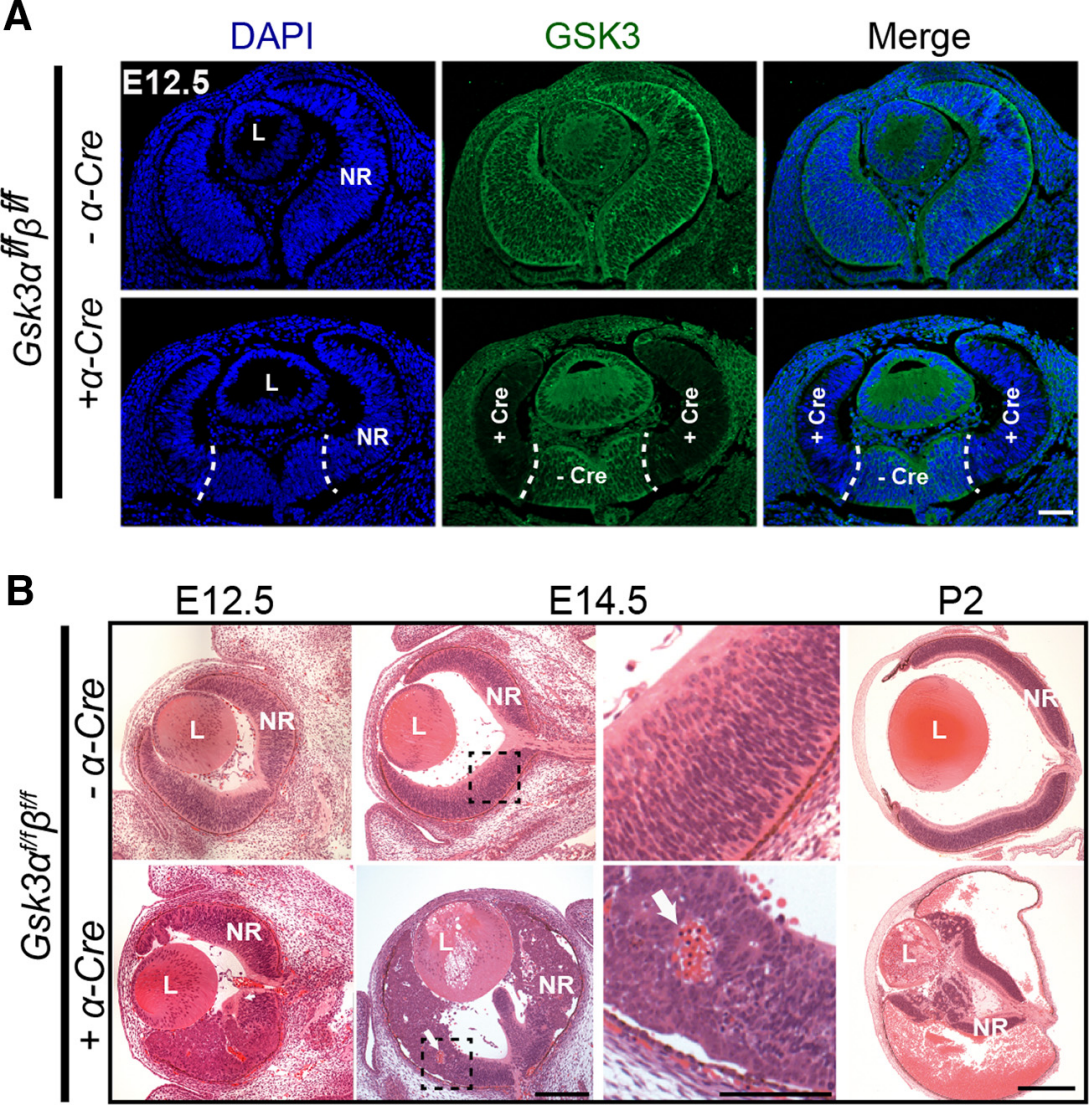
Developmental defects and microphthalmia in *Gsk3*-deficient retina with aberrant nuclear translocation of β-catenin, a key effector of the Wnt canonical pathway. ***A***, IHC of E12.5 retinas from *Gsk3α^f/f^β^f/f^* mice expressing or not α-*Cre* using a pan-GSK3 antibody (green) shows efficient deletion at the periphery where the *Cre* expression has been previously reported (delimited by dashed line). Scale bar, 100 μm. ***B***, H&E staining on methacrylate sections at E12.5, E14.5, and P2 reveals large retinal morphogenesis defects in *Gsk3α^f/f^β^f/f^*;*α-Cre* with blood invasion into the eyeball (showed by white arrow). L, Lens; NR, neural retina. Scale bars: E12.5 and E14.5, 100 μm; P2, 500 μm; E14.5, right, magnified image, 50 μm.

We crossed the floxed *Gsk3α^f/f^β^f/f^* mice with *α-Cre* (*αPax6-Cre*) line to generate *Gsk3α^f/f^β^f/f^;α-Cre* mice in which *Gsk*3 deletion occurs only in retinal progenitors as early as E10.5 (Marquardt et al., 2001). We first validated our model by assessing the efficacy of *Gsk3α* and *Gsk3β* deletion at E12.5. IHC using an antibody recognizing both GSK3 proteins showed ubiquitous expression in control retinas (Fig. 1*A*). Both *Gsk3* genes were efficiently deleted in the peripheral retina of *Gsk3α^f/f^β^f/f^;α-Cre* mice, but their expression in the central retina remained preserved consistent with the previously described *α-Cre* expression pattern (Marquardt et al., 2001).

H&E staining revealed major morphologic defects with profound retinal disorganization, including the loss of radial arrangement as well as folds and aggregates of RPCs, in *Gsk3α^f/f^β^f/f^;α-Cre* retina as early as E12.5 (Fig. 1*B*). In addition, blood was detected inside the retinal neuroblastic layer. The structure of the retina worsened rapidly during development, although the central part remained unperturbed, consistent with continued *Gsk3* expression in this region. At and after E14.5, the retina was largely reduced, whereas the eye size itself was comparable to those of littermate controls (Fig. 1*B*). A large quantity of blood accumulated inside the eyeball at P2. Finally, growth of the eyeball was severely reduced, leading to microphthalmia in the adult (data not shown).

### Multiple allelic combinations revealed functional redundancy of *Gsk3α* and *Gsk3β* in retinal development

**Figure 2.**
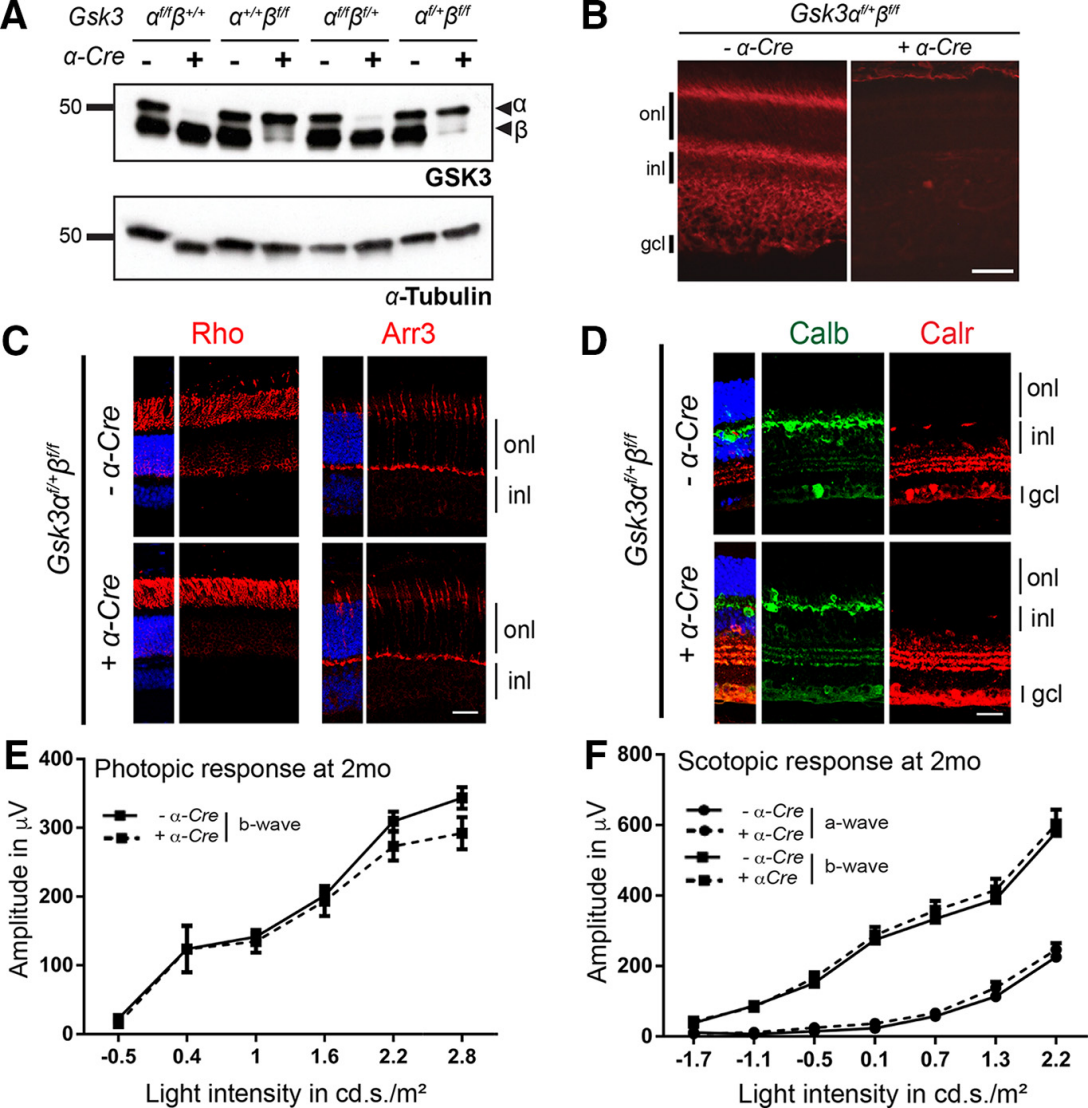
One allele of either *Gsk3α* or *Gsk3β* is sufficient for the development of a functional retina. ***A***, Immunoblot analysis of protein extracts from 2-month-old animals with a different combination of *Gsk3α* and *Gsk3β* floxed alleles (*Gsk3α^f/f^β^+/+^*, *Gsk3α^+/+^β^f/f^*, *Gsk3α^f/+^β^f/f^*, or *Gsk3α^f/f^β^f/+^*) with or without Cre recombinase using anti-pan GSK3 antibody (recognizing both isoforms) reveals decreased expression of GSK3α or GSK3β (arrowheads). *α*-Tubulin is used as a loading control. ***B***, IHC on 2-month-old retinal sections from control and *Gsk3α^f/+^β^f/f^*; *α-Cre* retinas with or without Cre recombinase using anti-GSK3β antibody (red) showing ubiquitous *Gsk3β* expression in all retinal layers, whereas its expression is lost in the Cre-expressing retina. ***C***, Expression of only one *Gsk3* allele (*Gsk3α*) is sufficient for proper photoreceptor development. IHC using anti-rhodopsin (Rho; red) and anti-Cone arrestin (Arr3; red) antibodies to label rod and cone photoreceptors, respectively. ***D***, Expression of only one *Gsk3* allele (*Gsk3α*) is sufficient for proper interneuron development. IHC using anti-Calretinin (Calr; green) and anti-calbindin (Calb; red) antibodies to label horizontal and amacrine cells, respectively. onl, outer nuclear layer; inl, inner nuclear layer; gcl, ganglion cell layer. Scale bar, 20 μm. ***E***, ***F***, ERG recording in 2-month-old *Gsk3α^f/+^β^f/f^;α-Cre* animals and littermate controls. Photopic (cones; ***E***) and scotopic (rods; ***F***) responses in *Gsk3α^f/+^β^f/f^;α-Cre* animals are similar to those in controls. The mean ± SEM intensity response curves of a- and b-wave responses were averaged from eight biological replicates of each genotype.

Severe deleterious effects by the loss of both *Gsk3α* and *Gsk3β* in RPCs in early development preclude the analysis of late retinal histogenesis. To circumvent this, we generated animals with different combinations of *Gsk3* deletion (loss of only one *Gsk3* gene: *Gsk3α^f/f^β^+/+^;α-Cre* or *Gsk3α^+/+^β^f/f^;α-Cre*, or three-quarter deletion: *Gsk3α^f/f^β^f/+^;α-Cre* or *Gsk3α^f/+^β^f/f^;α-Cre*). Immunoblot analysis using anti-GSK3 antibody (recognizing both proteins) in 2-month-old animals with different combinations of *Gsk3α* and *Gsk3β* floxed alleles demonstrated the efficacy of *Gsk3α* and *Gsk3β* deletion in areas where the Cre recombinase was expressed during early retinal development (all retinal progenitors with the exception of a stripe located in the dorsocentral region (Marquardt et al., 2001; Fig. 2*A*). IHC analysis using anti-GSK3β showed ubiquitous expression of *Gsk3β* in adult control retina and its complete loss in *Gsk3α^f/+^β^f/f^;α-Cre* retina (Fig. 2*B*). At 2 months, retinal histology revealed the correct laminated architecture with normal photoreceptors and interneurons when even one allele of *Gsk3α* (Fig. 2*C*,*D*) or *Gsk3β* (data not shown) was present. Photopic and scotopic ERG recordings, corresponding to cone and rod function, respectively, did not show any significant difference between *Gsk3α^f/+^β^f/f^;α-Cre* and control retina (Fig. 2*E*,*F*). These results were similar in mice carrying any combination of *Gsk3* deletion (data not shown). We therefore conclude that a single allele of wild-type *Gsk3α* or *Gsk3β* is sufficient to rescue obvious structural and functional defects in the complete absence of GSK3 signaling.

### Loss of either *Gsk3α* or *Gsk3β* in RPCs leads to increased number of displaced retinal ganglion cells

**Figure 3.**
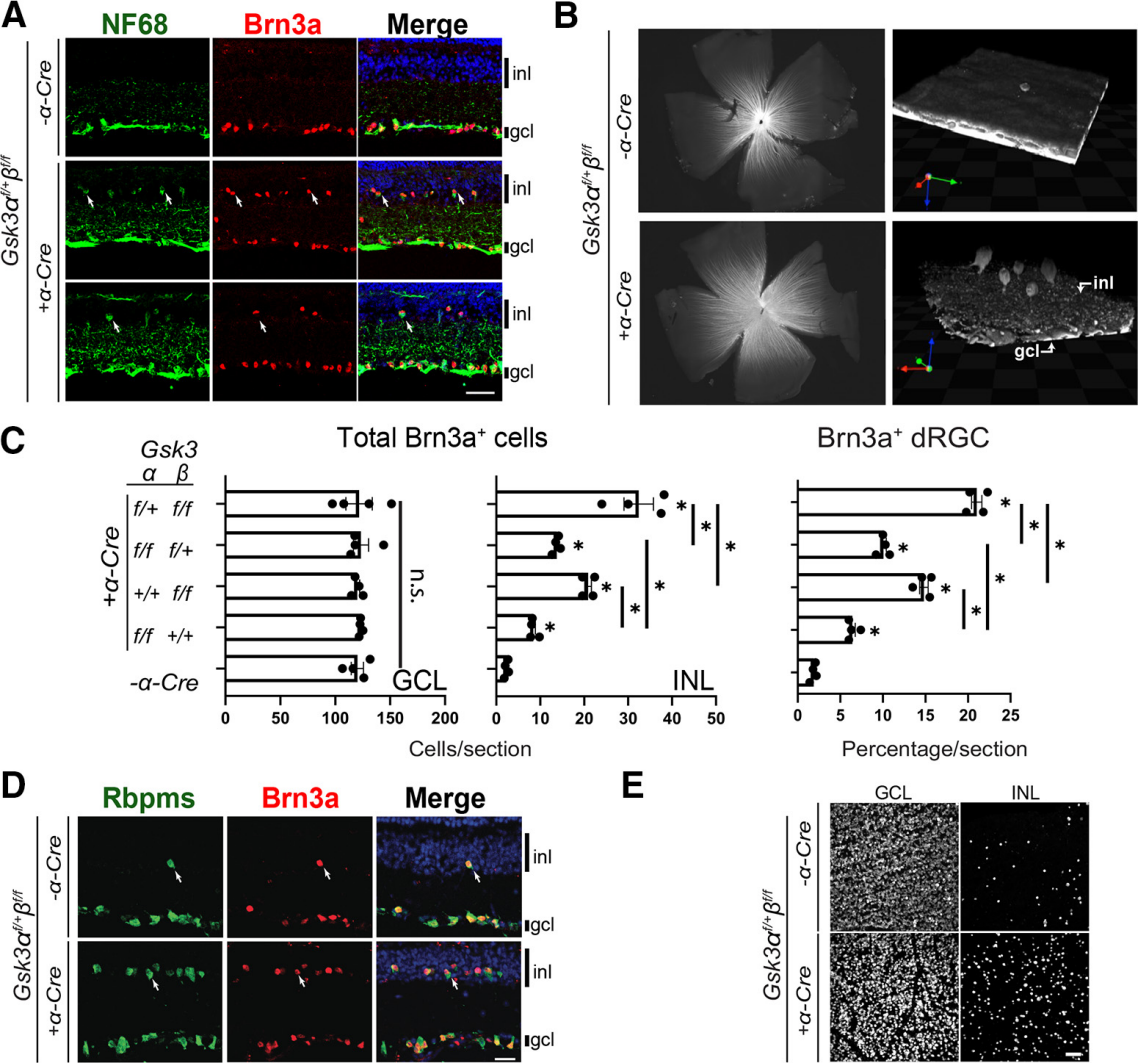
Gradual loss of *Gsk3α* and/or *Gsk3β* leads to an increased number of Brn3a-positive retinal ganglion cells displaced in the INL of adult retina. ***A***, Brn3a (red) and NF68 (green) IHC on 2-month-old *Gsk3α^f/+^β^f/f^; α-Cre* mouse retinas reveals the presence of supernumerary dRGCs (arrows) in the INL of *Gsk3α^f/+^β^f/f^; α-Cre* compared with littermate controls. Top panel represents control retinas, middle panel represents a peripheral retinal area, and bottom panel represents a more central area. Scale bar, 20 μm. ***B***, dRGCs send their axons into the optic nerve. Visualization of dRGCs after 3D reconstruction of 2-month-old flat mounted retinas of control and *Gsk3α^f/+^β^f/f^*; *α-Cre* animals following retrograde labeling with rhodamine-dextran applied onto the optic nerve. inl: inner nuclear layer, gcl: ganglion cell layer. ***C***, Gradual loss of *Gsk3α* and *Gsk3β* alleles (*Gsk3α^f/f^β^+/+^, Gsk3α^+/+^β^f/f^*, *Gsk3α^f/+^β^f/f^* or *Gsk3α^f/f^β^f/+^*) leads to a gradual increase of Brn3a-positive RGCs located to the INL, with the highest number observed in *Gsk3α^f/+^β^f/f^*; *α-Cre* animals. Left histograms represent counting of the total number of Brn3a-positive cells per section located in the GCL (left) or in the INL (middle). Right histogram represents the percentage of the dRGCs among the total number of Brn3a-positive cells per section for each combination. Mean ± SEM values are presented from four biological replicates. A nonparametric Mann–Whitney *U* test was applied. *p ≤* *0.05; ns, non significant. ***D***, Brn3a (red) and Rbpms (green) IHC on 2-month-old mouse retinas reveal the coexpression of these two RGC markers (dRGCs, arrows) in the INL of both *Gsk3α^f/+^β^f/f^; α-Cre* dRGCs and in littermate controls. Scale bar, 20 μm. ***E***, Flat-mounted retinas from *Gsk3α^f/+^β^f/f^; α-Cre* and littermate controls labeled with anti-Rbpms antibody demonstrated the large number of Rbpms-positive dRGCs in the INL of *Gsk3α^f/+^β^f/f^; α-Cre* mice. Scale bar, 50 μm. See Extended Data Figure 3-1 for Islet1 and Rbpms colocalization used to complete dRGC characterization and the repartition of the dRGCs in the retina. See Extended Data Figure 3-2, showing that Brn3a-positive dRGCs are not positive for amacrine or horizontal cell markers.

Although a single allele of either *Gsk3α* or *Gsk3β* permitted normal retinal development (Fig. 2), we observed a striking increase in the number of RGCs, as indicated by Brn3a-positive cells, in the INL of *Gsk3α^f/+^β^f/f^;α-Cre* retina compared with controls (Fig. 3*A*). Brn3a-positive cells in the INL have been described as dRGCs, a rare cell type in the mammalian retina (Buhl et al., 1988; Doi et al., 1994). All Brn3a-positive cells in the INL of *Gsk3α^f/+^β^f/f^;α-Cre* retina also expressed NF68 that labels cell bodies and axons of RGCs (Fig. 3*A*). To validate that these Brn3a-positive cells in the INL were indeed RGCs with axonal projections included in the optic nerve, we performed retrograde labeling with rhodamine-dextran applied onto the optic nerve of *Gsk3α^f/+^β^f/f^;α-Cre* mice. Subsequent 3D reconstructions on flat-mounted retinas revealed the presence of numerous fluorescent cell bodies located in the INL compared with controls (Fig. 3*B*) demonstrating that axons of dRGCs indeed reached the optic nerve. Thus, Brn3a-positive cells located in the INL of *Gsk3α^f/+^β^f/f^;α-Cre* retinas are indeed RGCs.

10.1523/ENEURO.0171-21.2021.f3-1 Figure 3-1 dRGCs express the nuclear factor Islet-1. ***A***, IHC on 2-month-old mouse retina reveals that most dRGCs (Rbpms-positive dRGCs, white arrows, red) in the INL of *Gsk3α^f/+^β^f/f^; α-Cre* and littermate controls were positive for Islet-1 (green), a marker expressed in the nuclei of ganglion cells, and of cholinergic amacrine cells, ON-bipolar cells, and subpopulations of horizontal cells. onl, Outer nuclear layer; inl, inner nuclear layer; gcl, ganglion cell layer. Scale bar, 50 μm. ***B***, Counting on flat mount of Rbpms- or Brn3a- positive cells located in the INL at the dorsal, ventral, nasal, and temporal part of control and *Gsk3α^f/+^β^f/f^*;*α-Cre* retina. Histogram represents the number of Brn3a- or Rbpms-positive cells per field. Mean ± SEM values are presented from four biological replicates. Download Figure 3-1, TIF file.

10.1523/ENEURO.0171-21.2021.f3-2 Figure 3-2 Brn3a-positive cells located in the INL of *Gsk3α^f/+^β^f/f^*; *α-Cre* retina are dRGCs. Brn3a-positive RGCs located in the INL of *Gsk3α^f/+^β^f/f^; α-Cre* retina do not express markers of other INL neurons such as CHAT or calbindin (Calb). onl, Outer nuclear layer; inl, inner nuclear layer; gcl, ganglion cell layer. Arrowheads indicates Brn3a-positive dRGCs. Scale bar, 20 μm. Download Figure 3-2, TIF file.

Increased dRGCs were observed in retinas carrying any combination of *Gsk3* deletions tested (*Gsk3α^f/f^β^+/+^*, *Gsk3α^+/+^β^f/f^*, *Gsk3α^f/+^β^f/f^*, or *Gsk3α^f/f^β^f/+^*), with the highest number detected in *Gsk3α^f/+^β^f/f^;α-Cre* mice compared with controls (10-fold increase; Fig. 3*C*). To note, the *Gsk3α^f/+^β^f/+^;α-Cre* retina did not display an excess of dRGCs (data not shown). Interestingly, the increase in numbers of dRGCs is not associated with a significant reduction in the number of RGCs in the ganglion cell layer (GCL), referred to as orthotopic RGCs (oRGCs; Fig. 3*C*).

Because of their low number in control retina (∼2% of RGCs), dRGCs have been poorly characterized with very few markers identified, such as Brn3a (Nadal-Nicolás et al., 2012, 2014). Immunostaining on sections and flat-mounted retinas with additional RGC marker antibodies revealed that dRGCs in *Gsk3α^f/+^β^f/f^;α-Cre* retina were also positive for Rbpms (Rodriguez et al., 2014), confirming their increased number in the INL compared with controls (Fig. 3*D*,*E*). Similar results were observed with Islet1 labeling (Extended Data Fig. 3-1*A*; Bejarano-Escobar et al., 2015). Previous work showed that the number of dRGCs varies by retinal domain (Dräger and Olsen, 1980). Counting of Rbpms- or Brn3a-positive dRGCs on *Gsk3α^f/+^β^f/f^;α-Cre* flat-mounted retinas did not show any significant differences in their distribution among the dorsal, ventral, nasal, and temporal regions (Extended Data Fig. 3-1*B*). Finally, Brn3a-positive dRGCs did not express markers of other INL neurons such as choline-acetyltransferase (CHAT; amacrine cells) or calbindin (horizontal and amacrine cells; Extended Data Fig. 3-2). Altogether, our results strongly support a ganglion cell identity of the displaced Brn3a- and RPBMS-positive cells located in the INL of *Gsk3α^f/+^β^f/f^;α-Cre.*

**Figure 4.**
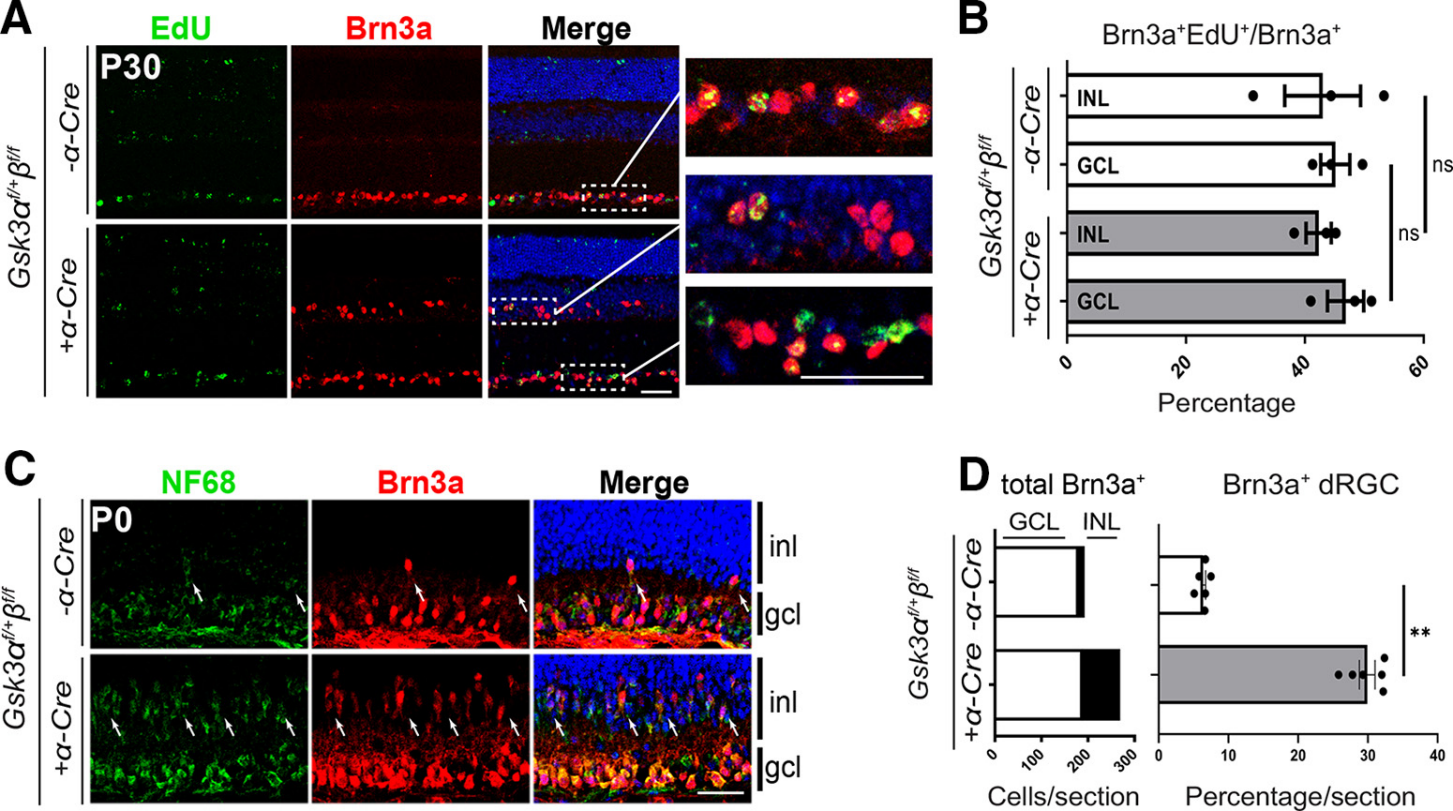
dRGCs are produced in the same differentiation wave as oRGC located in the GCL. ***A***, EdU-positive cells (green) and Brn3a-positive cells (red) were found both in the GCL and the INL of 30-day-old *Gsk3α^f/+^β^f/f^*; *α-Cre* animals after a single injection of EdU at E12.5. ***B***, Percentage of EdU- and Brn3a-positive cells located either in the GCL or in the INL among total number of Brn3a-positive cells. Mean ± SEM values are presented from three to four biological replicates. A nonparametric Mann–Whitney *U* test was applied. ns, non significant. ***C***, Brn3a (red) and NF68 (green) immunostaining on P0 mouse retinas revealed that a large number of dRGCs were already present in *Gsk3α^f/+^β^f/f^; α-Cre* mice, but were fewer in littermate controls (white arrows). ***D***, Left stacked histogram represents the counting of the total number of Brn3a-positive cells per section located in the GCL (white bars) and the INL (black bars) of *Gsk3α^f/+^β^f/f^; α-Cre* retinas. Right histogram represents the percentage of the dRGCs among the total number of Brn3a-positive cells per section. Mean ± SEM values are presented from six biological replicates. A nonparametric Mann–Whitney *U* test was applied. **p ≤* *0.01. inl, inner nuclear layer; gcl, ganglion cell layer. Scale bar, 20 μm.

To test whether dRGCs in *Gsk3* mutant mice were produced during the same developmental time window as oRGCs, we performed pulse chase experiments by injecting EdU at E12.5, at the peak of RGC birth. Retinal sections from 1-month-old animals were then immunolabeled using anti-Brn3a antibody (Fig. 4*A*). In control and *Gsk3α^f/+^β^f/f^*;*α-Cre* retina, we identified 40–50% of RGCs that were Brn3a/EdU-positive in all layers examined (GCL and INL), indicating that both dRGCs and oRGCs were born around the same time (Fig. 4*B*). We next examined whether dRGCs are overproduced during normal retinal development and eliminated later on. In this context, increased numbers of dRGCs in *Gsk3α^f/+^β^f/f^*;*α-Cre* retinas could result from a defect in dRGC riddance occurring during the first 2 postnatal weeks, a period of developmental cell death in the retina (Young, 1984; Galli-Resta, 1996). At P0, the number of Brn3a-positive oRGCs was similar between littermate control and *Gsk3α^f/+^β^f/f^*;*α-Cre* retinas (Fig. 4*C*,*D*). In contrast, the proportion of Brn3a-positive cells located in the inner part of neuroblastic layer, corresponding presumably to dRGCs, was much lower in control retinas (6 ± 0.1%) compared with *Gsk3α^f/+^β^f/f^*;*α-Cre* retinas (30 ± 1.4%). Thus, dRGCs are not overproduced and eliminated postnatally in control retina. Our results demonstrate that dRGCs are generated during early waves of retinogenesis in *Gsk3α^f/+^β^f/f^*;*α-Cre* retina and strongly suggest that GSK3s play a role in restricting their numbers during normal retinal development.

### dRGCs produced in the absence of either *Gsk3α* or *Gsk3β* project to accessory visual system circuitry

**Figure 5.**
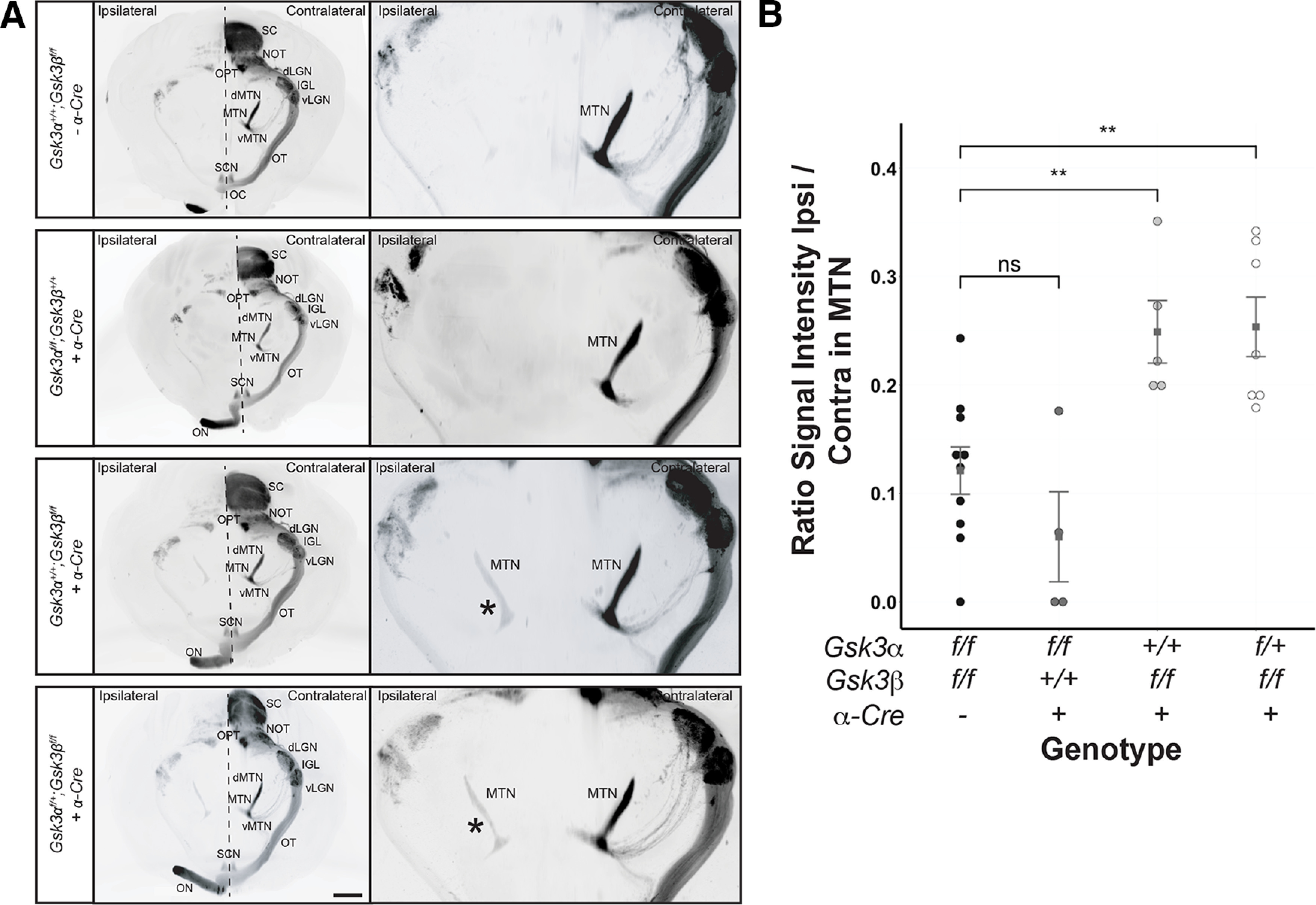
Lack of Gsk3β results in RGC projections into the ipsilateral medial terminal nucleus. ***A***, All panels are light sheet fluorescence microscopy of solvent-cleared adult brain from control, *Gsk3α^f/f^β^+/+^; α-Cre*, *Gsk3α^+/+^β^f/f^; α-Cre*, and *Gsk3α^f/+^β^f/f^; α-Cre* animals after intravitreal injection of CTB coupled to either Alexa Fluor-555 or Alexa Fluor-647. Ipsilateral projections of RGCs into the MTN were observed in the absence of *Gsk3β* expression. NOT, Nucleus of optic tract; LGN, lateral geniculate nucleus; vLGN, ventral LGN; IGL, intergeniculate leaflet; OPT, olivary pretectal nucleus; MTN, medial terminal nucleus; dMTN, dorsal MTN; vMTN, ventral MTN; OT, optic tract; SCN, suprachiasmatic nucleus; ON, optic nerve; SC, superior colliculus. Scale bar, 1 mm. *Ipsilateral MTN. ***B***, Quantification of the signal intensity ratio between ipsilateral and contralateral MTN in controls and *Gsk3* mutants (including *Gsk3α^f/f^β^+/+^; α-Cre*, *Gsk3α^+/+^β^f/f^; α-Cre*, and *Gsk3α^f/+^β^f/f^; α-Cre*). A nonparametric Mann–Whitney *U* test was applied. ns, non significant. **p ≤* *0.01. See Extended Data Figure 5-1 for costaining of the CTB-positive cells with Brn3a and NF68.

Previous studies in birds and reptiles have reported that dRGCs could be responsible for optokinetic nystagmus, as they mostly project to the accessory optic nuclei (AOSs; Cook and Podugolnikova, 2001), which are critical for non-image-forming circuit and image stabilization (Simpson, 1984). To test whether dRGCs in *Gsk3* mutants project into specific visual nuclei in the brain, including the AOS, we traced the total pool of RGCs, including dRGCs, with CTB. Bilateral injection of CTB, coupled to either an Alexa Fluor-555 or Alexa Fluor-647 followed by 3D imaging, allowed us to trace both ipsilateral and contralateral projecting axons. We first confirmed that CTB injections indeed marked the dRGCs based on flat-mounts of retinas after Brn3a and NF68 immunolabeling (Extended Data Fig. 5-1). To visualize the entire visual projection network, we conducted whole-brain clearing using iDISCO+ followed by light-sheet fluorescent imaging and 3D reconstruction (Fig. 5*A*). The complete loss of *Gsk3β* yielded a large increase in ipsilateral projecting RGCs in one of the three nuclei composing the AOS, the MTN (Simpson, 1984). This terminal nucleus is the main component of the AOS, reacting best to either upward or downward movement and mediating the optokinetic nystagmus, which is critical for image stabilization (Yonehara et al., 2009). Calculation of the signal intensity ratio between the ipsilateral and contralateral MTN demonstrated a significant increase in RGC projections into the ipsilateral MTN in retinas with *Gsk3β* deletion (Fig. 5*B*). This result suggests that excess dRGCs might participate in the non-image-forming circuit.

10.1523/ENEURO.0171-21.2021.f5-1 Figure 5-1 Intravitreal injection of CTB labels dRGCs. After intravitreal injection of CTB coupled to an Alexa Fluor-555 (red) in *Gsk3α^f/+^β^f/f^; α-Cre* eye led to the labeling of Brn3a-positive (green) and NF68-positive (gray) cells located in the INL. Scale bars, 20 μm. Download Figure 5-1, TIF file.

### Whole-transcriptome analysis suggests that dRGCs in GSK3 mutant retinas are direction‐selective ganglion cells

**Figure 6.**
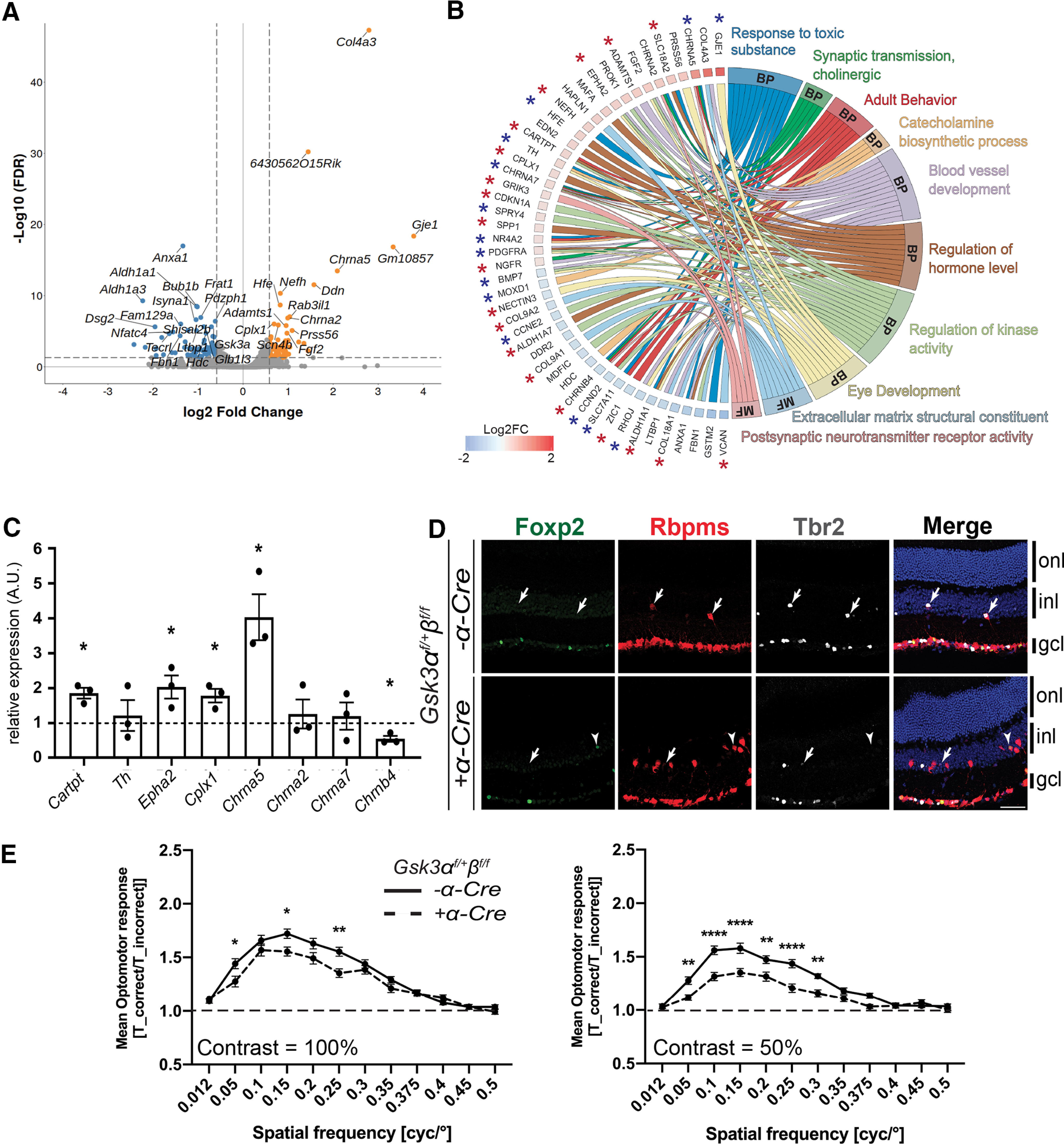
Whole-transcriptome meta-analysis suggests that dRGCs in the *Gsk3α^f/+^β^f/f^*; *α-Cre* retina are DS-RGCs. ***A***, Volcano plot representation of differentially expressed genes between *Gsk3α^f/+^β^f/f^; α-Cre* and control retinas plotted on the *x*-axis (log2 scale). FDR-adjusted significance is plotted on the *y*-axis. Orange and blue dots indicate significantly upregulated and downregulated genes in *Gsk3α^f/+^β^f/f^; α-Cre* retinas, respectively. Vertical dashed lines represent FC = 1.5. Horizontal dashed line represents FDR = 0.05. ***B***, Chord plot representation of DEGs related to GO (Gene Ontology) annotations belonging to either molecular function (MF) or biological process (BP). Overlaps in GO annotation among genes within each category are visualized. *Genes expressed in previously published purified RGCs (blue, slightly expressed genes in RGCs between 1 and 5 FPKM; red, highly expressed genes in RGCs >5 FPKM). ***C***, qRT-PCR validation of selected DEGs identified by RNA-Seq analysis. Differential expression analysis by qRT-PCR of *Cartpt*, *Th*, *Epha2*, *Cplx1*, *Chrna5*, *Chrna2*, *Chrna7*, and *Chrnb4* in *Gsk3α^f/+^β^f/f^; α-Cre* retinas at 2 months of age, relative to levels in littermate control retinas. All values are expressed as the mean ± SEM from three biological replicates. A nonparametric Mann–Whitney *U* test was applied, *p ≤* *0.05. ***D***, IHC on 2-month-old mouse retinas reveals the presence of a subset of dRGCs (Rbpms-positive dRGCs, red) in *Gsk3α^f/+^β^f/f^; α-Cre* expressing either the transcription factor Tbr2 (gray) or Foxp2 (green). Arrows indicate Tbr2 and Rbpms-positive dRGCs; arrowheads represent Foxp2 and Rbpms-positive dRGCs. onl, outer nuclear layer; inl, inner nuclear layer; gcl, ganglion cell layer. Scale bar, 50 μm. ***E***, The mean OMR indices (±SEM) are plotted as a function of spatial frequency for each genotype (*n* = 13 for *Gsk3α^f/+^β^f/f^* and 18 for *Gsk3α^f/+^β^f/f^*;*α-Cre* genotype). The baseline (1; dashed line) represents unspecific head movements and no response to the stimulus. OMR at 100% and 50% contrast in *Gsk3α^f/+^β^f/f^; α-Cre* mice (dashed line) and controls (black line). A Grubbs’ test was performed at 5% to remove outliers followed by two-way ANOVA: *p ≤* *0.05, **p ≤* *0.01, ***p ≤* *0.001. Extended Data Figure 6-1 for the hierarchical clustering of the DEGs. See Extended Data Figure 6-2 for pathway analysis results.

We next performed transcriptome analysis using RNA-Seq to identify molecular changes in adult *Gsk3α^f/+^β^f/f^*;*α-Cre* retina and to better characterize dRGCs. Retinas from *Gsk3α^f/+^β^f/f^* mice were used as controls. Gene-level analysis revealed 111 DEGs using filtering criteria of FC = 1.5, with an FDR cutoff of ≤0.05 and a minimum mean expression value of 1 FPKM in at least one of the two experimental groups (Fig. 6*A*, Extended Data Fig. 6-1). Pathway analysis of DEGs revealed several statistically significant overrepresented pathways (Extended Data Fig. 6-2). Biological processes and molecular function pathways included 48 DEGs; of these, 33 genes were expressed in RGCs based on published whole-transcriptomic data from purified RGCs (for a total 69 RGC-expressed genes among the 111 DEGs; see Fig. 8*B*, stars; Sajgo et al., 2017). The dominance of RGC-expressed genes in our dataset is consistent with the high number of dRGCs observed in the *Gsk3α^f/+^β^f/f^*;*α-Cre* retina.

10.1523/ENEURO.0171-21.2021.f6-1 Figure 6-1 Hierarchical clustering of the identified differentially expressed genes. Hierarchical clustering representing the 111 DEGs [abs(FC), ≥1.5; FDR, ≤0.05; FPKM, >1] between 2-month-old *Gsk3α^f/+^β^f/f^; α-Cre* retina and those of littermate controls were clustered by their *z*-score. Each column for each genotype corresponds to one sample. For both groups, triplicates were analyzed. Left, Downregulated genes; Right, upregulated genes. Download Figure 6-1, TIF file.

10.1523/ENEURO.0171-21.2021.f6-2 Figure 6-2 Identification of enriched pathways from DEGs identified in 2-month-old *Gsk3α^f/+^β^f/f^; α-Cre* retina. ***A***, Gene ontology (GO) annotations of DEGs in *Gsk3α^f/+^β^f/f^; α-Cre* retinas compared with those in littermate controls. Top over-represented pathways for biological process (BP), molecular function (MF), KEGG (Kyoto Encyclopedia of Genes and Genomes), and TRRUST (transcriptional regulatory relationships unrevealed by sentence-based text mining) were identified by enrichment analysis using Metascape. ***B***, Circular visualization for BP and MF of GO enrichment analysis. Downregulated genes (blue dots) and upregulated genes (red dots) within each GO pathway are plotted based on logFC. The *z*-score bars indicate whether an entire GO category is more likely to be increased or decreased based on the genes within it. Download Figure 6-2, TIF file.

Among interesting candidates dysregulated in the biological processes and molecular function pathways (Fig. 6*B*, Extended Data Fig. 6-2), we identified *Chrna2*, *Chrna5*, *Chrna7*, and *Chrnb4* encoding for postsynaptic subunits of the nicotinic cholinergic receptor. With the exception of *Chrna2*, all other genes are upregulated in *Gsk3α^f/+^β^f/f^*;*α-Cre* retina. Most retinal ganglion cells express nicotinic receptors (Kay et al., 2011; Rousso et al., 2016). Among other potentially relevant genes, the *Grik3* gene product belongs to the kainate family of glutamate receptors functioning as ligand-activated ion channels. In direction‐selective ganglion cells (DSGCs), glutamate is proposed to be the main source of excitation (Sweeney et al., 2014, 2019). Finally, *Cartpt*, encoding for the preprotein CART (Cocaine- And Amphetamine-Regulated Transcript Protein) that was upregulated in *Gsk3α^f/+^β^f/f^*;*α-Cre* retina, was validated by qRT-PCR (Fig. 6*C*). *Cartpt* is specifically expressed in direction-selective RGCs (DS-RGCs; Rousso et al., 2016), suggesting that dRGCs (or at least a subset) in the *Gsk3α^f/+^β^f/f^*;*α-Cre* retina might be DS-RGCs. In support of this hypothesis, we found some dRGCs in *Gsk3α^f/+^β^f/f^*;*α-Cre* and littermate control retinas that were positive for the transcription factor Tbr2, which has been described as being essential for RGC specification participating in non-image-forming visual circuits (Fig. 6*D*; Simpson, 1984; Yonehara et al., 2009). A small subset of dRGCs also expressed Foxp2, a transcription factor involved in DS-RGC differentiation in mice (Fig. 6*D*; Sato et al., 2017). These two factors were expressed in a mutually exclusive way in Rpbms-positive dRGCs, suggesting that dRGCs in *Gsk3α^f/+^β^f/f^*;*α-Cre* might encompass several subtypes.

### Optomotor response is impaired in GSK3 mutant

Given that DS-RGCs are reported to drive the OMR by projecting mainly into the contralateral AOS (Simpson, 1984; Yonehara et al., 2009), we tested the OMR of *Gsk3α^f/+^β^f/f^*;*α-Cre* mice. The OMR indices (*T*_correct/*T*_incorrect) were calculated from three trials at contrasts 100% and 50% (Fig. 6*E*). At 100% contrast, the OMR indices were significantly reduced in *Gsk3α^f/+^β^f/f^*;*α-Cre* mice compared with controls at 0.05, 0.15, and 0.25 cpd. The maximum OMR index was observed at 0.15 cpd in controls, whereas it reached a maximum at 0.1 in *Gsk3α^f/+^β^f/f^*;*α-Cre* mice. At 50% contrast, the OMR indices were also significantly reduced in *Gsk3α^f/+^β^f/f^*;*α-Cre* mice compared with controls, but to a larger extent between 0.05 and 0.3 cpd. The maximum OMR index was observed at 0.15 cpd in both controls and *Gsk3α^f/+^β^f/f^*;*α-Cre* mice. Altogether, these results demonstrate an impaired OMR in *Gsk3α^f/+^β^f/f^*;*α-Cre* mice. These data, together with our transcriptomic and axonal projection analyses, suggest that at least a subset of dRGCs expressing only one allele of *Gsk3α* are DS-RGCs.

## Discussion

In this study, we report that complete loss of GSK3 in retinal progenitors leads to microphthalmia in adult mice with severe morphological defects. Such a severe phenotype was not observed anymore when only one *Gsk3α* or *Gsk3β* allele was expressed, confirming the functional redundancy of these two genes. Our results implicate GSK3s as the first reported determinants of dRGCs during retinal histogenesis. Indeed, we show that mouse retinas with only one allele of *Gsk3* exhibit an excessive number of dRGCs. The concomitant large increase of axonal projections to the ipsilateral MTN, our RNA-Seq data, and optomotor response tests, have led us to propose that these dRGCs are involved in the detection of image motion direction.

In pigmented wild-type mouse retina, dRGCs in the INL are a very rare and poorly described type of cell, which represents only 2% of RGCs (Balkema and DrÄger, 1990; Doi et al., 1994; Dräger and Olsen, 1980; Nadal-Nicolás et al., 2014). It is therefore striking that dRGC numbers increase up to 20% of RGCs when a single copy of *Gsk3α* is present in retinal progenitors. To our knowledge, such a high number of dRGCs has never been reported in a transgenic/mutant animal. A previous study hypothesized that dRGCs are misplaced in the INL because of an ontogenic aberration rather than representing an independent class of RGCs (Buhl and Dann, 1988; Doi et al., 1994). Indeed, differential cell adhesion plays a key role in the sorting and migration of retinal cells in their appropriate layers, especially for RGCs. One can therefore hypothesize that enhanced dRGCs in mice with a single copy of *Gsk3* is the consequence of increased aberration events. This hypothesis could be supported by our RNA-Seq data showing the upregulation of genes coding for collagen subunits (*Col18a1*, *Col4a3*, *Col9a1*, and *Col9a2*) and extracellular matrix proteins in the *Gsk3α^f/+^β^f/f^*;*α-Cre* retina, which could favor migration defects. Noticeably, if it were the case, the increase in dRGCs should be accompanied by a decrease in oRGCs. However, we found that the number of oRGCs in the GCL is unaltered, strongly suggesting that RGCs in the INL of mice with a single copy of *Gsk3* represent a specific subtype of RGCs. In support of this, topographic and quantitative analyses of RGCs in albinos and pigmented rats indicate that dRGCs are not misplaced by ontogenic mistakes but indeed represent a specific subpopulation of RGCs (Nadal-Nicolás et al., 2014). GSK3β was previously shown to be involved in neural cell fate decision by controlling the timing of the activity of bHLH transcription factors, such as NeuroD or Neurog2 (Moore et al., 2002; Li et al., 2012). If dRGCs are not produced following ontogenic aberrations but are instead determined by a proper genetic program, it would be interesting to identify the transcription factors involved and seek for any regulation by GSK3s. Along this line, further studies would allow a better understanding of whether the excess of dRGCs occurs only because of an expanded pool of normally occurring dRGCs or whether their presence is also a consequence of an aberrant migration during retinal development when GSK3s are not fully active. New sequencing technology such as single-cell RNA-Seq would definitively be an asset to shed more light on specific markers for dRGCs and to identify key players of dRGC specification/differentiation. As a distinct cell type, scRNA-Seq analysis following high-depth sequencing should highlight a cell cluster in t-distributed stochastic neighbor embedding plots in retina with only one *Gsk3α* allele expressed corresponding to dRGCs. The interest and power of such approach has already been demonstrated for RGC characterization (Rheaume et al., 2018). Whole-transcriptome analysis at early time points when RGCs are produced might also complete such analysis.

In reptiles, amphibians, and birds, only dRGCs project into the MTN, whereas in mammals only oRGCs have been reported as projecting into the MTN (Fite et al., 1981; Dann and Buhl, 1987; Krause et al., 2014). Our results obtained from anterograde labeling clearly demonstrated a large increase in ipsilateral MTN projections in the absence of *Gsk3β*, whereas it was absent or very dim in control animals. Although this strongly suggests that excess dRGCs in mutant mice are causing this phenotype, we cannot exclude the possibility that mutant oRGCs also participate in these ipsilateral MTN projections. However, contralateral projections did not seem to be affected. Noticeably, however, it would be challenging to observe an increase in dRGC projections into the other areas already strongly labeled using our anterograde labeling method, especially into the dorsal lateral geniculate nucleus (dLGN) or superior colliculus (SC). We can speculate that the low number of ipsilateral MTN projections in the control condition reflects the low number of dRGCs present in the WT retina and could therefore explain why such a result had not been described so far. Altogether, our results strongly suggest that dRGCs may primarily project into the ipsilateral MTN. In mice, it has been shown by retrograde labeling from the SC, which receives a large number of RGC projections, that dRGCs/oRGCs project to one or both SCs (Karten et al., 1977). Although challenging, similar experiments (i.e. fluorescent dye injection into the ipsilateral MTN) may allow us to discriminate whether the increased signal in the absence of *Gsk3β* originates only from dRGCs and whether these cells also project into this area in WT retina. In regard to our results, it is still unclear whether the function of GSK3 is to limit the number of dRGCs and to actively regulate their correct projection to the contralateral MTN or whether GSK3 function is limited to tightly controlling the number of dRGCs, which project thereafter to the ipsilateral MTN in a GSK3-independent manner.

Given the very low percentage of dRGCs in the control retina, their function is poorly studied in mammals. In contrast, dRGC function, projections, and topography have been investigated in bird and reptile retina (Mouritsen et al., 2004). In birds, cryptochrome-expressing dRGCs are used as a magnetic compass for orientation (Nießner et al., 2016). In European Robin birds, *Erithacus rubecula*, a low number of dRGCs has been identified but specifically express Cryptochrome 1b only during nocturnal migration period (Nießner et al., 2016). In rodents, retrograde labeling from the optic nerve led to the identification of 16 classes of dRGCs based on the ramification levels of their dendrites as well as the dendritic field size (Pang and Wu, 2011). Based on dRGC dendrite projections into the inner plexiform layer, it has been proposed that most dRGCs in the WT retina are functionally more involved in retinal OFF light pathways (Pang and Wu, 2011). Similar methods applied to the *Gsk3α^f/+^β^f/f^*;*α-Cre* retina should shed more light on dRGC function and establish whether all the different classes are present.

As part of the AOS, the MTN receives an afferent signal from the eye and sends an efferent signal to motor neurons controlling the position of the eye. As such, optokinetic reflex relies on direction-specific retinal projections to the AOS. Neurons of the dorsal terminal nucleus code for horizontal stimulus, whereas neurons of the MTN code for vertical stimulus (Giolli et al., 2006; Yonehara et al., 2009). Therefore, the direction of image motion relies on DS-RGCs in the retina. The alteration of the OMR in *Gsk3α^f/+^β^f/f^*;*α-Cre* mice support the hypothesis that some of the supernumerary dRGCs are indeed related to motion detection. Although the number of dRGCs was drastically increased, the OMR was not increased but, on the contrary, was reduced. Such a result might be caused by the higher number of projections to the ipsilateral side instead of the contralateral one, leading to an alteration of the neuronal circuit regulating the OMR (Simpson, 1984; Yonehara et al., 2009). We also identified in *Gsk3α^f/+^β^f/f^*;*α-Cre* and control retinas a small subset of dRGCs, which are positive for the transcription factors Tbr2 and Foxp2, the markers for non-image-forming RGCs and DS-RGCs, respectively (Nadal-Nicolás et al., 2014). Together with the transcriptomic data (upregulation of genes such as *Cartpt* expressed in DS-RGCs), these results provide strong evidence suggesting that the large number of dRGCs in the *Gsk3α^f/+^β^f/f^*;*α-Cre* retina might indeed be DS-RGCs projecting into the MTN. It has been recently proposed that dRGCs might also be involved in predator detection by integrating overhead visual information (Nadal-Nicolás et al., 2014). Using suitable and complementary visual tests, our genetic model could be highly valuable to complete the functional identification of the dRGCs in visual process.

Overall, our results demonstrate a critical role of GSK3s in stringently regulating the number of a rare type of dRGCs, which has been poorly described so far. *Gsk3* mutant mice, with a large number of dRGCs in their retina, offer a unique and powerful model system to further study the embryonic origin, synaptic connections, and visual function of dRGCs in mammals.

## Synthesis

Reviewing Editor: Siegrid Löwel, University of Goettingen Decisions are customarily a result of the Reviewing Editor and the peer reviewers coming together and discussing their recommendations until a consensus is reached. When revisions are invited, a fact-based synthesis statement explaining their decision and outlining what is needed to prepare a revision will be listed below. The following reviewer(s) agreed to reveal their identity: Carol Schuurmans. Note: If this manuscript was transferred from JNeurosci and a decision was made to accept the manuscript without peer review, a brief statement to this effect will instead be what is listed below.

In this manuscript, the authors provide new insights into the regulation of RGC development that contribute to our understanding of the nature of displaced retinal ganglion cells and to the function of important signaling molecules.

The authors first examined the effects of Gsk3double knock-outs (DKOs) on retinal development (Figures 1-3) and then studied compound KOs in which only one wild-type allele is present, either Gsk3α or Gsk3β. In this second part of the manuscript, the authors ascribe a role for Gsk3α or Gsk3β in limiting the formation of displaced RGCs (dRGCs). They further perform transcriptomic and functional analyses in animals carrying one Gsk3 allele to further characterize the dRGCs, and to begin to elucidate mechanisms of action. The second part of the manuscript is generally better described and could on its own make a more coherent story.

For this manuscript to be of publishable quality, three relatively simple edits should be performed:

1. Figures 2 and 3 studies are incomplete and poorly connected to the rest of the manuscript and could be removed without detracting from the rest. Alternatively, additional experimentation would be required as outlined below.

2. The authors need to temper their conclusion that the dRGCs observed in the animals with one GSK3 allele are an expanded pool of normally occurring dRGCs vs aberrantly migrating dRGCs. They have a balanced discussion of these two viewpoints in the Discussion section. However, the title and abstract overstate their bias to one of the alternatives for which they do not have definitive proof. We suggest to simply temper their conclusions by changing the title and revising the abstract. The authors should also provide a statistical analysis of total RGC counts in Figure 4B as indicated, as the total RGC counts are an important piece of data that help to decipher the two possibilities (although not definitive on their own - it could simply be that more RGCs are made due to a progenitor cell fate choice that goes awry, and then separately, that these cells migrate aberrantly because of a lack of GSK3 activity at 100% levels).

In fact, it is surprising that there is not a difference in D/V/N/T number of dRGCs in the system because alpha Cre does not target the dorsal retina very efficiently, apart from a smattering of cells. It usually makes for a good internal control. This is interesting to know- maybe these rare targeted cells are dRGCs. dRGCs have a very strong bias along the DVNT plane of the mouse retina and are highly concentrated in the nasal peripheral retina. “Data not shown” is not sufficient to address this important issue: oversampling of the nasal retina would produce a result of higher dRGCs. The authors should show this especially because dRGCs have a low DVT number vs. very high nasal number and the Cre they are using does not delete in the DV retina, and presumably they are not counting in this region. This is a concern and a point of great interest- for example does their genetic manipulation cause the retina overall to take on the ventral number of dRGCs?

3. Along these lines: Several times the authors write ‘data not shown’ and then make conclusions based on this data. As highlighted in the detailed comments (below), the authors need to provide any data used to make conclusions, e.g. in the supplemental material.

General comments

1. While there is some nice, high quality data in this manuscript, we have several issues with the first part of the manuscript examining DKOs, which is not complete and poorly linked to the rest of the manuscript. In our view, this part of the manuscript should be removed, or truncated, and possibly only used to provide a rationale for studying embryos carrying one GSK3a or Gsk3b allele. Otherwise, a significant amount of work would be required to bring this part of the manuscript up to a publishable form. Our recommendation is to start this manuscript with Figure 1A,B that simply describes the severe morphological defects observed in DKO retinas, as a way to set the stage for the need to focus on embryos carrying a single GSK3a or Gsk3b allele. The initial study of DKOs could then be completed as a separate manuscript.

2. If the authors focus their manuscript on the animals carrying one allele of GSK3a or Gsk3b, they need to temper their conclusions. While it is appropriate to call the ectopically located Brn3a cells displaced RGCs, the direct comparison to naturally occurring dRGCs goes too far and should be toned down, including in the title and abstract. The authors have a much more balanced discussion of the two competing possibilities - that the reduction in GSK3 signaling either results in ontogenetic migratory defects or the respecification of a set of RGCs to a distinct dRGC fate, in the Discussion. However, their argument for favoring the distinct dRGC fate is only based on there being more dRGCs than oRGCs in GSK3 compound mutants, which in our view, is not a definitive argument, or strong enough evidence to support the claim.

If dRGCs have a distinct identity, they should have a unique transcriptome/epigenome, and not just a unique location in the retina. To directly relate GSK3 compound mutant dRGCs to those dRGCs that are naturally occurring, additional analyses would be needed. For example, scRNA-seq for population stratification of wild-type and mutant retinas. If dRGCs are indeed a distinct cell type, and sequencing is deep enough, they should be visible as a distinct cell cluster in tSNE plots. The authors may search published data sets to test this assumption and add these references to the discussion. The authors could also then discuss that in GSK3 compound mutants, if there is indeed an expansion of naturally occurring dRGCs, this expansion would be distinguishable in scRNA-seq data sets. In our view, this would provide more definitive proof of their assumption.

To be clear, we are not suggesting more experimentation, just a more balanced set of conclusions that is appropriate to the current data.

3. What is lacking in this study is a model of how the loss of GSK3 influences the fate and/or migratory properties of RGCs. The authors perform RNA-seq, but unfortunately, the outcomes of this study do not really help to clarify how the loss of GSK3 influences RGC development, except to say that a wide host of genes expressed in RGCs are deregulated.

In the Discussion, the authors suggest that “GSK3 may exerts its effects through phosphorylation of keys proteins involved in neural development, including proneural factors such as Neurogenin 2 and NeuroD (Li et al., 62 2012; Moore et al., 2002).” If this is the case, are any of the known targets of these bHLH TFs deregulated in the GSK3 mutants? Some additional analyses of the RNAs-seq data in comparison to other published data sets on Neurod1 and/or Neurog2 mutants, for example, would be informative.

Specific comments:

As mentioned above, we would recommend to remove Figures 2 and 3 and develop further for another study. Otherwise, there are several issues as described below.

Figure 2

• Researchers generally count EdU-labeled and pHH3-labeled cells to assess how many RPCs are cycling. In general, when there are more EdU+ RPCs, pHH3+ cells are also increased. It is therefore difficult to understand why there might be an increase in pHH3 cells at E12.5, while EdU is unchanged. Are cells accumulating in G2/M? Is there some sort of cell cycle pausing phenotype? Additional studies would be required to address these questions and provide further supportive evidence, such as flow cytometric profiling of labeled DNA, cumulative EdU labeling to measure the lengths of each cell cycle phase, etc.

• Mitoses generally occur on the apical side of the retina due to interkinetic nuclear migration. The ectopic pHH3 staining in GSK3 DKOs suggests that this process has gone awry, and mitoses now occur in the central retina. However, I am not entirely convinced that the pHH3 staining is real and not just background staining. Other measures of cell cycle kinetics would be required to provide further supportive data to indicated that there is an increase in G2/M phase cells in DKOs.

• The 16-hr time point after EdU administration is a bit long, as it will label cells that were in S-phase and have since left S-phase (4-6 hr long generally), entered G2/M (∼2 hr long), and then divided and re-entered G1. If there is more EdU/pHH3 labeling in GSK3 DKOs, this suggests some sort of G2 pausing has occurred. The authors simply claim that there are aberrant cell cycle kinetics, but this is not helpful, and they should think more deeply about what the data means. And additional studies are required to sort out what is actually going on, as indicated above

• In general, if the authors wish to label only S-phase RPCs, animals should be sacrificed 30 min after EdU administration to make sure labeled RPCs are still in S-phase at the time of analysis.

• If the authors wish to sort out the general question of how many RPCs are proliferating, they may consider other markers that label all cell cycle phases, such as Ki67

Figure 3/S1B.

• On pg. 3 lines 149-152 the authors say: “However, by E14.5, Hes1 positive cells were sparser at the periphery where Gsk3 expression was absent (Figure S1B). In the same area lacking Gsk3 expression, other progenitor markers, such as Pax6 and Sox2, were similarly decreased in Gsk3αf/fβf/f;a-Cre retina compared to controls (data not shown).”

• Firstly, they should show the Pax6 and Sox2 data

• Secondly, we are not sure how to reconcile the finding of a loss of RPC markers, and yet there are still dividing cells in the neural retina at E14.5 in Figure 2. If there are proliferating cells, what are they, if not RPCs?

• Thirdly, the authors show that there is a loss of RGC markers, and conclude as a Figure 3 title: “ Lack of Gsk3a and Gsk3b expression in retinal progenitors blocks retinal progenitor differentiation.” However, there is a big mass of cells in the DKO periphery, and if they are not RGCs and not RPCs, what are they? One possibility is that the RPCs have instead differentiated into another cell type. The authors would need to apply several markers of differentiated cells to identify the fate of the remaining cells in the DKO retinas.

Comments on the remainder of the study

Figure 4

• In the discussion, the authors make the claim that the dRGCs are likely to be the previously identified rare dRGCs with a unique identity based on their observation that there is an increase in total RGCs in GSK3 compound KOs, and that this increase is solely due to the increase in dRGCs and not RGCs located in the GCL. However, the graph in Figure 4B has no statistical analyses, s.e.m.s, or individual data points, so it is not clear whether there are significant changes in total RGCs or not. This data should be analysed.

• The authors state: “Finally, Brn3a-positive dRGCs did not express markers of other INL neurons such as Choline-Acetyltransferase (CHAT, amacrine cells), Calretinin (amacrine cells), or Calbindin (horizontal cells) further confirming their RGC identity (data not shown).” This data should be shown.

•

Figure 5

• The authors state: “We first confirmed that CTB injections indeed marked the dRGCs based on flat mounts of retinas after Brn3a immunolabelling (data not shown).” This data should be shown.

## Author Response

We would like to thank the reviewers for their positive feedbacks and their suggestions. Below is a point-by point reply to all their comments that we addressed in our revised version of the manuscript.

1. Figures 2 and 3 studies are incomplete and poorly connected to the rest of the manuscript and could be removed without detracting from the rest. Alternatively, additional experimentation would be required as outlined below.

We agree with the reviewers that the first part of the manuscript could be removed to focus the manuscript on the mouse models with partial loss of Gsk3a and Gsk3b displaying increased number of dRGCs. Therefore, all the parts of the manuscript concerning the DCKO have been removed accordingly.

2. The authors need to temper their conclusion that the dRGCs observed in the animals with one GSK3 allele are an expanded pool of normally occurring dRGCs vs aberrantly migrating dRGCs. They have a balanced discussion of these two viewpoints in the Discussion section. However, the title and abstract overstate their bias to one of the alternatives for which they do not have definitive proof. We suggest to simply temper their conclusions by changing the title and revising the abstract. The authors should also provide a statistical analysis of total RGC counts in Figure 4B as indicated, as the total RGC counts are an important piece of data that help to decipher the two possibilities (although not definitive on their own - it could simply be that more RGCs are made due to a progenitor cell fate choice that goes awry, and then separately, that these cells migrate aberrantly because of a lack of GSK3 activity at

100% levels).

We partially agree with the reviewer and we modified the title and the abstract accordingly. We removed the part referring to the subtype in the title. Based on our data showed in this manuscript and as mentioned by the reviewer, we clearly demonstrated the presence of an excess of dRGCs. Although we cannot completely rule out that this excess of dRGCs is due to an aberrant migration during development, we showed several lines of evidence indicating that they are originating from an expanded pool of normally occurring dRGCs. We added the following sentence at the end of the second paragraph to complete the discussion concerning this point. “Along this line, further studies would allow to better understand if the excess of dRGCs is only due to an expanded pool of normally occurring dRGCs or if their presence is also a consequence of an aberrant migration during retinal development when GSK3s are not fully active.

As requested, we now provide statistics in Figure 3C for the number of ganglion cells in the GCL and in the INL. We do not observe a significant reduction in the number of RGCs in the GCL when GSK3 is partially removed.

In fact, it is surprising that there is not a difference in D/V/N/T number of dRGCs in the system because alpha Cre does not target the dorsal retina very efficiently, apart from a smattering of cells. It usually makes for a good internal control. This is interesting to know- maybe these rare targeted cells are dRGCs. dRGCs have a very strong bias along the DVNT plane of the mouse retina and are highly concentrated in the nasal peripheral retina. “Data not shown” is not sufficient to address this important issue: oversampling of the nasal retina would produce a result of higher dRGCs. The authors should show this especially because dRGCs have a low DVT number vs. very high nasal number and the Cre they are using does not delete in the DV retina, and presumably they are not counting in this region. This is a concern and a point of great interest- for example does their genetic manipulation cause the retina overall to take on the ventral number of dRGCs?

We agree with the reviewers that it is surprising that there are no differences in the different quadrants of the retina in our controls with no increased number of dRGCs in the temporal side (not nasal) as described by Drager and Olsen 1980 and Nadal-Nicholas et al 2014. In the first paper, they identified the excess of dRGC in the temporal side in pigmented and albino mice after retrograde labelling from the brain. In the second paper, they used immunolabelling on pigmented and albino rat retina. In our

study, we immunolabelled mouse retina from a mixed background (C57Bl6/129) which might explain the lack of differences. We did count the most peripheral part of each quadrant but the number of dRGCs in control was extremely low anyway. Concerning the CRE-positive retina, we would like to emphasize that we did not count specifically this narrow part of the retina where the CRE was not expressed. Again, we did not find a difference in the temporal side of the CRE-positive retina compared to other quadrants. In this context, it would suggest GSK3 function during retinal development is to regulate dRGC production during retinal development.

3. Along these lines: Several times the authors write ‘data not shown’ and then make conclusions based on this data. As highlighted in the detailed comments (below), the authors need to provide any data used to make conclusions, e.g. in the supplemental material.

We agreed on this comment and added more supplementary data to support our conclusion (cf details

after each specific comment related to this point)

General comments

1. While there is some nice, high quality data in this manuscript, we have several issues with the first part of the manuscript examining DKOs, which is not complete and poorly linked to the rest of the manuscript. In our view, this part of the manuscript should be removed, or truncated, and possibly only used to provide a rationale for studying embryos carrying one GSK3a or Gsk3b allele. Otherwise, a significant amount of work would be required to bring this part of the manuscript up to a publishable form. Our recommendation is to start this manuscript with Figure 1A,B that simply describes the severe morphological defects observed in DKO retinas, as a way to set the stage for the need to focus on embryos carrying a single GSK3a or Gsk3b allele. The initial study of DKOs could then be completed as a separate manuscript.

We thank the reviewer for his/her suggestion to publish the DCKO data in a separate manuscript. We therefore left in the revised version, as suggested, only previous Fig. 1A,B.

2. If the authors focus their manuscript on the animals carrying one allele of GSK3a or Gsk3b, they need to temper their conclusions. While it is appropriate to call the ectopically located Brn3a cells displaced RGCs, the direct comparison to naturally occurring dRGCs goes too far and should be toned down, including in the title and abstract. The authors have a much more balanced discussion of the two competing possibilities - that the reduction in GSK3 signaling either results in ontogenetic migratory defects or the respecification of a set of RGCs to a distinct dRGC fate, in the Discussion. However, their argument for favoring the distinct dRGC fate is only based on there being more dRGCs than oRGCs in GSK3 compound mutants, which in our view, is not a definitive argument, or strong enough evidence to support the claim.

We partially agree with the reviewer comment and we modified the title and abstract accordingly to be more balanced. (cf previous response to this point)

If dRGCs have a distinct identity, they should have a unique transcriptome/epigenome, and not just a unique location in the retina. To directly relate GSK3 compound mutant dRGCs to those dRGCs that are naturally occurring, additional analyses would be needed. For example, scRNA-seq for population stratification of wild-type and mutant retinas. If dRGCs are indeed a distinct cell type, and sequencing is deep enough, they should be visible as a distinct cell cluster in tSNE plots. The authors may search published data sets to test this assumption and add these references to the discussion. The authors could also then discuss that in GSK3 compound mutants, if there is indeed an expansion of naturally

occurring dRGCs, this expansion would be distinguishable in scRNA-seq data sets. In our view, this would provide more definitive proof of their assumption.

We completely agree with the reviewer that scRNA-seq with sufficient depth of sequencing could shade more light on a specific transcriptome for dRGCs if they are a distinct cell type. As suggested, we looked at scRNA-seq data from Rheaume et al, 2018 and looked at the distribution of our DEGs across all clusters. Unfortunately, they are spread in many different clusters and it was not informative. It is not surprising regarding the low number of dRGCs in WT retina and the lack of identified specific markers. Of note, around 10 DEGs were found in their scRNA-seq data. Despite the fact that the comparison did not help much to further the analysis of our RNA-seq data, we added the interest of scRNA-seq data in the discussion.

We edited the beginning of the third paragraph of the discussion to discuss this point. “New sequencing technology such as single cell RNA-Seq would definitively be an asset to shed more light on specific markers for dRGCs and to identify key players of dRGC specification/differentiation. As a distinct cell type, scRNA-seq analysis following high depth sequencing should highlight a distinct cell cluster in tSNE plots in retina with only one Gsk3a allele expressed corresponding to dRGCs. The interest and power of such approach has already been demonstrated for RGC characterization (Rheaume et al., 2018) “.

To be clear, we are not suggesting more experimentation, just a more balanced set of conclusions that is appropriate to the current data.

We thank the reviewer for the constructive comments.

3. What is lacking in this study is a model of how the loss of GSK3 influences the fate and/or migratory properties of RGCs. The authors perform RNA-seq, but unfortunately, the outcomes of this study do not really help to clarify how the loss of GSK3 influences RGC development, except to say that a wide host of genes expressed in RGCs are deregulated.

In the Discussion, the authors suggest that “GSK3 may exerts its effects through phosphorylation of keys proteins involved in neural development, including proneural factors such as Neurogenin 2 and NeuroD (Li et al., 62 2012; Moore et al., 2002).” If this is the case, are any of the known targets of these bHLH TFs deregulated in the GSK3 mutants? Some additional analyses of the RNAs-seq data in comparison to other published data sets on Neurod1 and/or Neurog2 mutants, for example, would be informative.

We chose to perform RNA-Seq analysis in adult retina therefore it is difficult to compare our data with other data available online when they were done during retinal development. However, we compared our data to single cell data from RGCs (Rheaume et al, 2018) as mentioned in a previous response to the reviewer.

We also looked at NeuroD1 KO expression data in the retina (Ochocinska et al, 2013) but they are microarray data from 2-months old animals where NeuroD1 has been deleted in photoreceptor precursors using Crx-Cre. Therefore, this dataset does not bring more information to our study. We also looked at Neurog2 KO RNA-Seq data performed at P2 (Kowalchuk et al. 2018) and found only two genes in common with our RNA-Seq data: Moxd1 and Zic1. Again, the time points being different, such comparison is not really informative. However, repeating RNA-seq data at early time points to identify putative factors involved in dRGC genesis should be perform in further studies. To address reviewers’ comment, we added one line at the end of the second paragraph of the discussion. “Whole transcriptome analysis at early time points when RGCs are produced might also complete such analysis"

Specific comments:

As mentioned above, we would recommend to remove Figures 2 and 3 and develop further for another study. Otherwise, there are several issues as described below.

As suggested, we removed Figures 2 and 3 and modified the manuscript accordingly. Comments on the remainder of the study

Figure 4

• In the discussion, the authors make the claim that the dRGCs are likely to be the previously identified rare dRGCs with a unique identity based on their observation that there is an increase in total RGCs in GSK3 compound KOs, and that this increase is solely due to the increase in dRGCs and not RGCs located in the GCL. However, the graph in Figure 4B has no statistical analyses, s.e.m.s, or individual data points, so it is not clear whether there are significant changes in total RGCs or not. This data should be analysed.

The graphs have been replaced to show the number of RGCs in the GCL and in the INL with the mean and SEM. Statistics have been done and no significant differences are observed in the GCL.

• The authors state: “Finally, Brn3a-positive dRGCs did not express markers of other INL neurons such as Choline-Acetyltransferase (CHAT, amacrine cells), Calretinin (amacrine cells), or Calbindin (horizontal cells) further confirming their RGC identity (data not shown).” This data should be shown.

As requested by the reviewer, we now provide a supplementary figure (Figure 3-2) and modified the sentence related to the figure in the manuscript. In this new figure, we show that dRGCs are not positive for Chat (Amacrin cell marker) or Calbindin (Horizontal and Amacrin cell marker). However, after repeating Calretinin staining, we found few Brn3a-positive oRGCs and dRGCs that are also positive for this marker that we did not noticed previously. Such colocalization has been described in other papers (Haverkamp et al 2000; Lee et al 2018; Lee et al 2010.). Therefore, we did not include the staining with calretinin because it was not informative for our purpose to specifically label amacrine cells. We updated this part of the manuscript: “Finally, Brn3a-positive dRGCs did not express markers of other INL neurons such as Choline-Acetyltransferase (CHAT, amacrine cells) or Calbindin (horizontal and amacrine cells) (Figure 3-2). Altogether, our results strongly support the ganglion cell identity of the displaced Brn3a- and RPBMS-positive cells located in the INL of Gsk3αf/+βf/f ; a-Cre.”

Figure 5

• The authors state: “We first confirmed that CTB injections indeed marked the dRGCs based on flat mounts of retinas after Brn3a immunolabelling (data not shown).” This data should be shown.

Figure 5-1 has been added to the revised manuscript showing the colocalization of the CTB-positive cells located in the INL with Brn3a and NF68 in Gsk3αf/+βf/f ; a-Cre.”Unfortunately, we were not able to take pictures of the control retina without a-Cre because of the very low number of dRGCs.

We have made changes as suggested, to respond to the points in Synthesis Statement and the reviewer’s comments. We hope that our revised manuscript is now
