# Supplementary figures and images for: Glycogen Synthase Kinase 3 Regulates the Genesis of Displaced Retinal Ganglion Cells3
Source: eNeuro. 2021 Oct 5;8(5):ENEURO.0171-21.2021. doi: 10.1523/ENEURO.0171-21.2021 (PMC8496207; doi:10.1523/ENEURO.0171-21.2021)

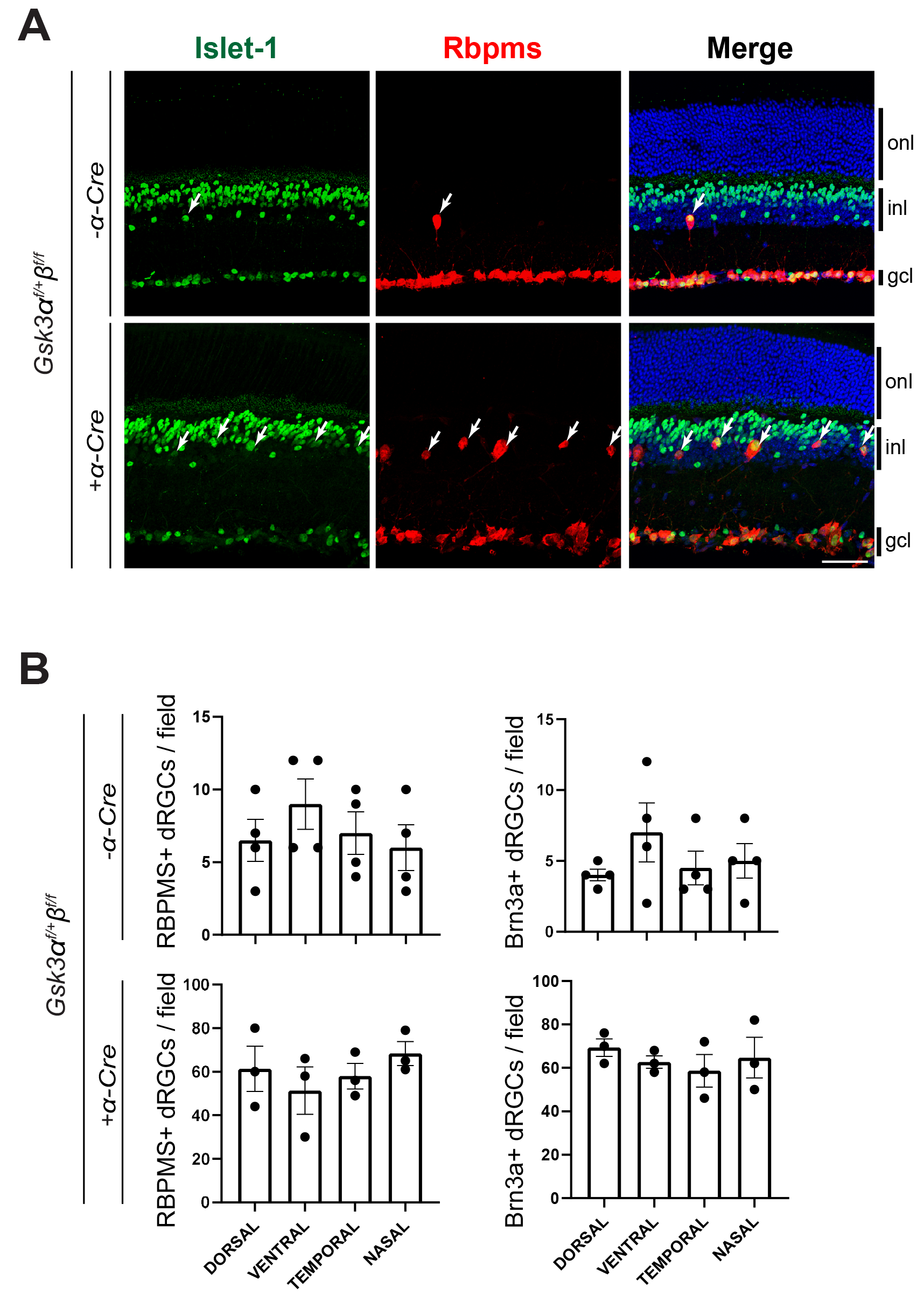

Supplement: Figure 3-1 — dRGCs express the nuclear factor Islet-1. A, IHC on 2-month-old mouse retina reveals that most dRGCs (Rbpms-positive dRGCs, white arrows, red) in the INL of Gsk3αf/+βf/f; α-Cre and littermate controls were positive for Islet-1 (green), a marker expressed in the nuclei of ganglion cells, and of cholinergic amacrine cells, ON-bipolar cells, and subpopulations of horizontal cells. onl, Outer nuclear layer; inl, inner nuclear layer; gcl, ganglion cell layer. Scale bar, 50 μm. B, Counting on flat mount of Rbpms- or Brn3a- positive cells located in the INL at the dorsal, ventral, nasal, and temporal part of control and Gsk3αf/+βf/f;α-Cre retina. Histogram represents the number of Brn3a- or Rbpms-positive cells per field. Mean ± SEM values are presented from four biological replicates. Download Figure 3-1, TIF file. [file enu-eN-NWR-0171-21-s02.tif]

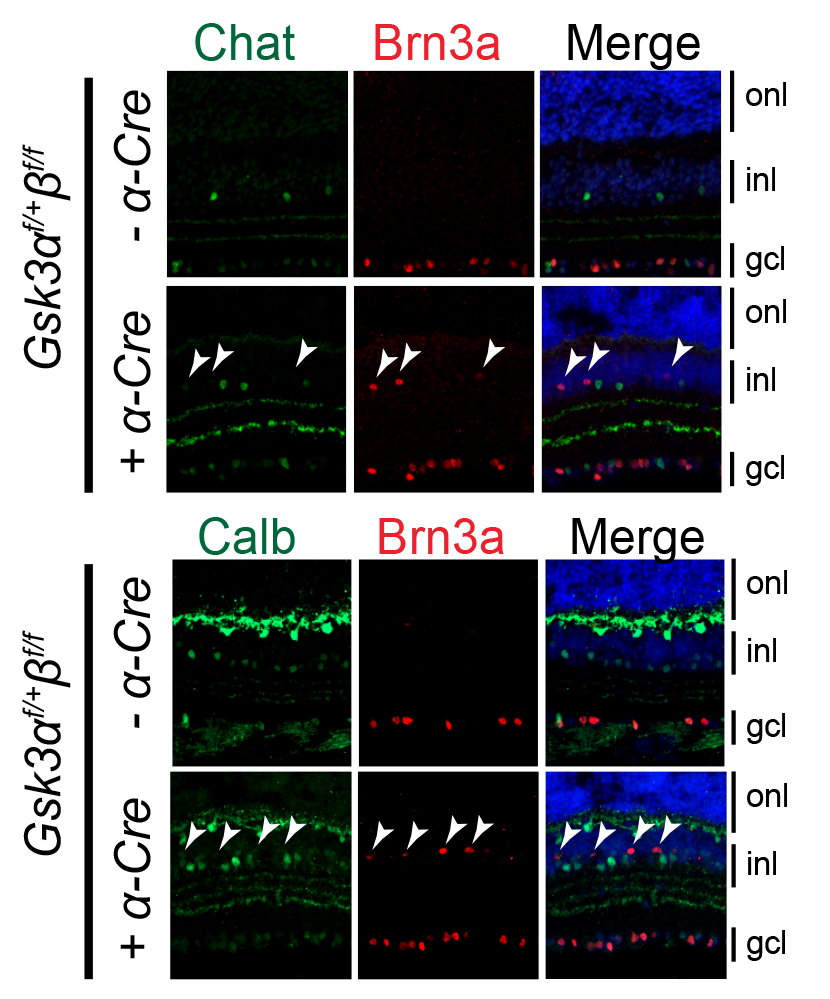

Supplement: Figure 3-2 — Brn3a-positive cells located in the INL of Gsk3αf/+βf/f; α-Cre retina are dRGCs. Brn3a-positive RGCs located in the INL of Gsk3αf/+βf/f; α-Cre retina do not express markers of other INL neurons such as CHAT or calbindin (Calb). onl, Outer nuclear layer; inl, inner nuclear layer; gcl, ganglion cell layer. Arrowheads indicates Brn3a-positive dRGCs. Scale bar, 20 μm. Download Figure 3-2, TIF file. [file enu-eN-NWR-0171-21-s03.tif]

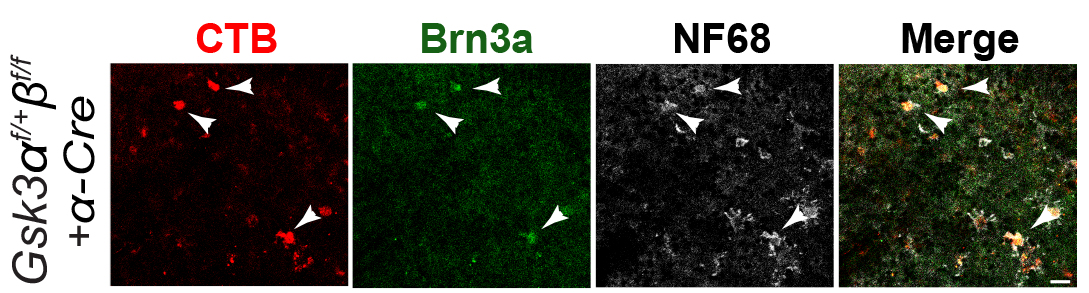

Supplement: Figure 5-1 — Intravitreal injection of CTB labels dRGCs. After intravitreal injection of CTB coupled to an Alexa Fluor-555 (red) in Gsk3αf/+βf/f; α-Cre eye led to the labeling of Brn3a-positive (green) and NF68-positive (gray) cells located in the INL. Scale bars, 20 μm. Download Figure 5-1, TIF file. [file enu-eN-NWR-0171-21-s04.tif]

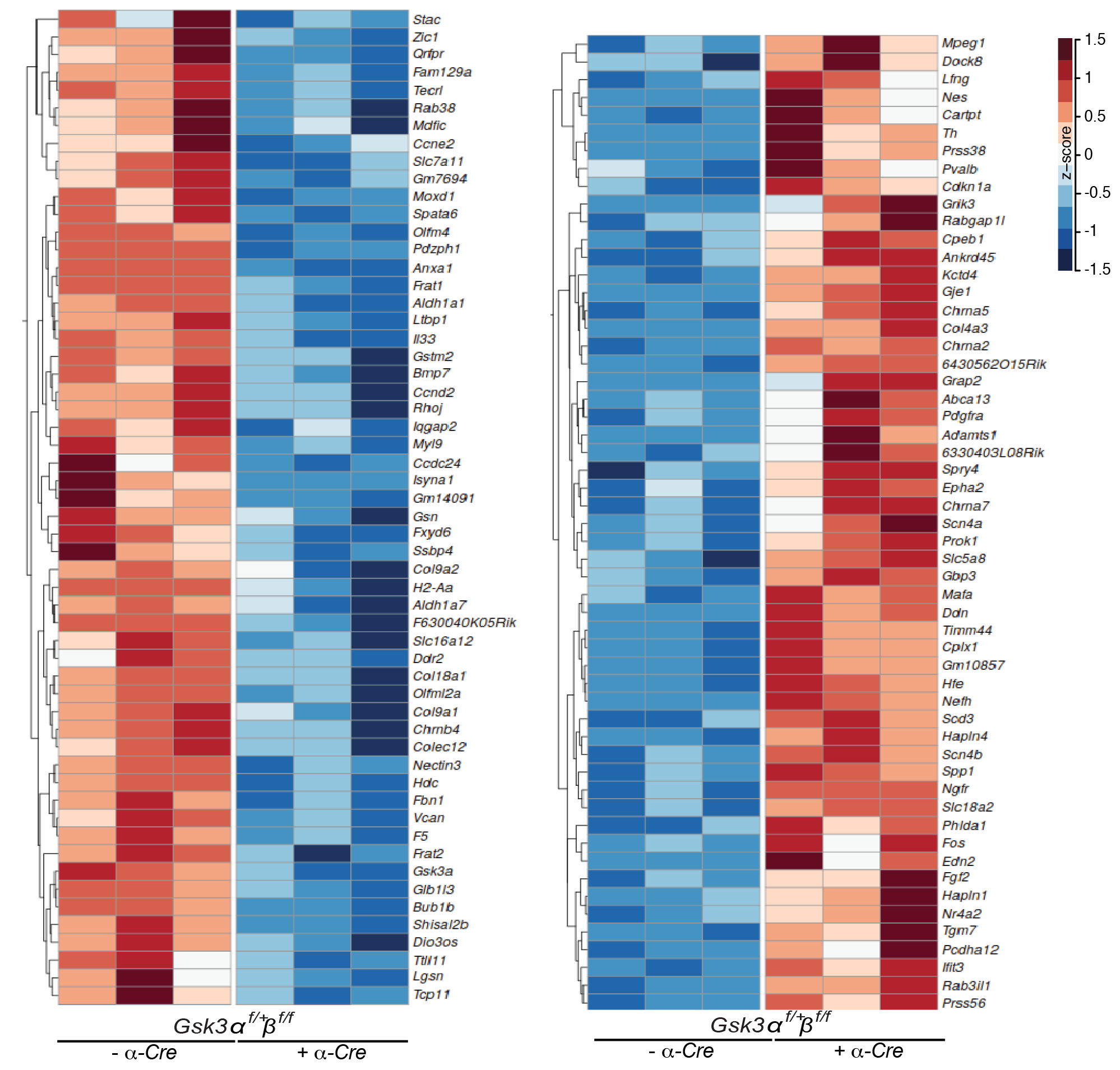

Supplement: Figure 6-1 — Hierarchical clustering of the identified differentially expressed genes. Hierarchical clustering representing the 111 DEGs [abs(FC), ≥1.5; FDR, ≤0.05; FPKM, >1] between 2-month-old Gsk3αf/+βf/f; α-Cre retina and those of littermate controls were clustered by their z-score. Each column for each genotype corresponds to one sample. For both groups, triplicates were analyzed. Left, Downregulated genes; Right, upregulated genes. Download Figure 6-1, TIF file. [file enu-eN-NWR-0171-21-s05.tif]

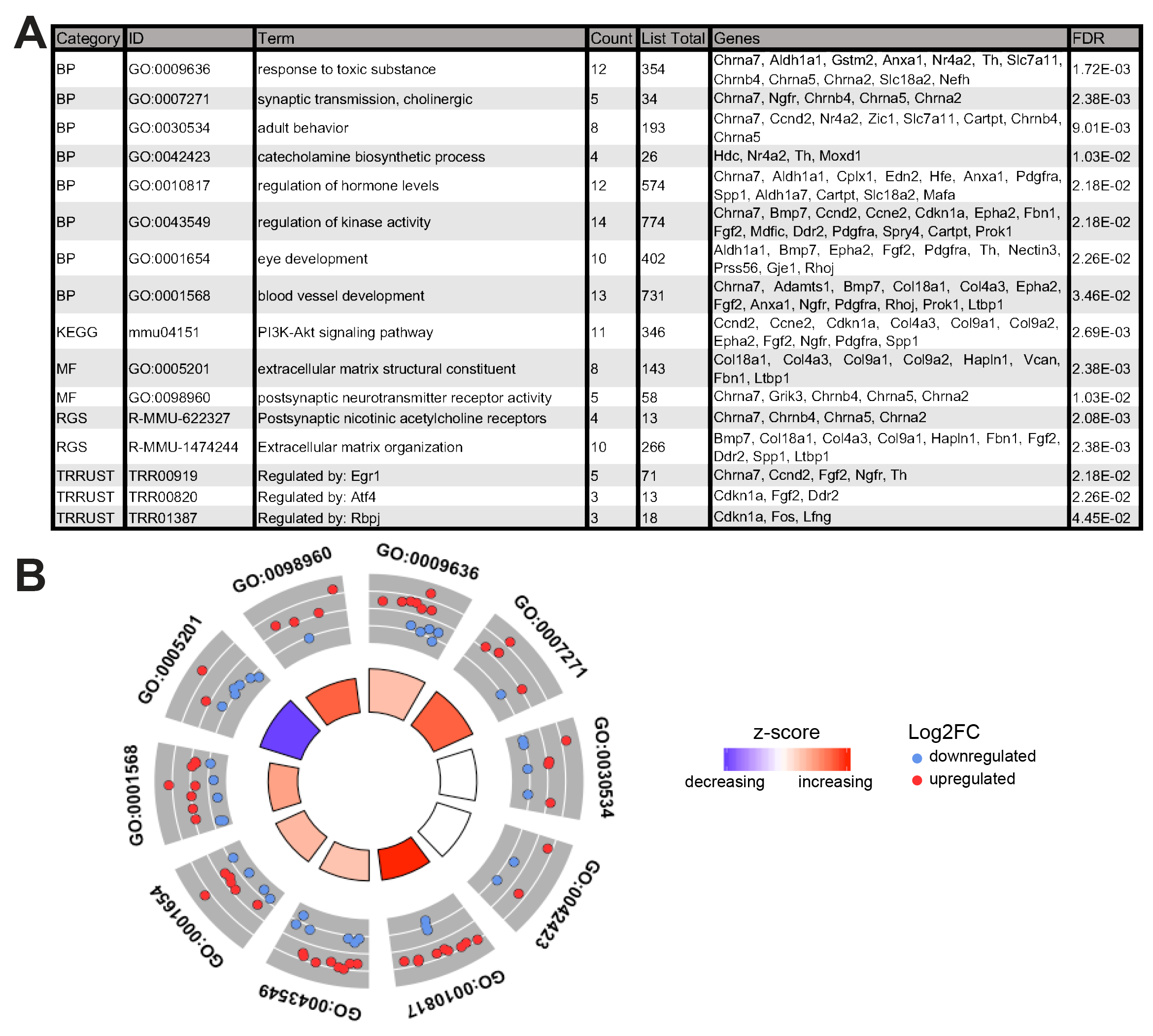

Supplement: Figure 6-2 — Identification of enriched pathways from DEGs identified in 2-month-old Gsk3αf/+βf/f; α-Cre retina. A, Gene ontology (GO) annotations of DEGs in Gsk3αf/+βf/f; α-Cre retinas compared with those in littermate controls. Top over-represented pathways for biological process (BP), molecular function (MF), KEGG (Kyoto Encyclopedia of Genes and Genomes), and TRRUST (transcriptional regulatory relationships unrevealed by sentence-based text mining) were identified by enrichment analysis using Metascape. B, Circular visualization for BP and MF of GO enrichment analysis. Downregulated genes (blue dots) and upregulated genes (red dots) within each GO pathway are plotted based on logFC. The z-score bars indicate whether an entire GO category is more likely to be increased or decreased based on the genes within it. Download Figure 6-2, TIF file. [file enu-eN-NWR-0171-21-s06.tif]
